# Adaptive Task Planning for Long-Horizon Robotic Manipulation Based on Video Priors and Dynamic Scene Graphs

**DOI:** 10.3390/s26175595

**Published:** 2026-09-03

**Authors:** Guanghui Ma, Jiahui Guo, Xinhua Tang, Huaidong Zhou, Yongfeng Rong

**Affiliations:** 1 School of Integrated Circuits, Anhui Polytechnic University, Wuhu 241000, China; maguanghui@ahpu.edu.cn; 2 School of Automation, Northwestern Polytechnical University, Xi’an 710072, China; guojiahui@mail.nwpu.edu.cn; 3 School of Artificial Intelligence, Shenzhen University, Shenzhen 518060, China; 18379498580@163.com; 4 Department of Computer Science and Technology, Tsinghua University, Beijing 100084, China; yfrong@tsinghua.edu.cn

**Keywords:** embodied intelligence, task planning, long-horizon robotic manipulation, vision-language model, video priors, dynamic scene graphs, Qwen3-VL, LIBERO-10

## Abstract

Robots are now expected to execute increasingly complex long-horizon tasks in unstructured environments. Despite the strong potential of pretrained Vision-Language Models (VLMs) in task planning, their direct application to robotic manipulation is hindered by logical reasoning deviations and inadequate geometric scene perception. This work proposes an adaptive task planning method based on video priors and dynamic scene graphs (ATP-VPDSG). It leverages the VLM to extract manipulation logic from video demonstrations, thus supplementing manipulation priors. Meanwhile, scene graphs were integrated to convert unstructured environments into structured representations with spatial topological relations, compensating for perceptual deficiencies. A dual-track feedback mechanism based on visual expectations was further incorporated to enable failure diagnosis and adaptive replanning in complex environments. Extensive long-horizon robotic manipulation experiments were conducted on the LIBERO-10 benchmark with Qwen3-VL as the core VLM. Results showed that ATP-VPDSG achieved an average task planning accuracy of 91.2% and a task execution success rate of 74.67%, outperforming the selected task planning baselines. Ablation studies verified that video priors and dynamic scene graphs exerted complementary effects on logical constraints and physical feasibility. Furthermore, a real-robot experiment on an industrial slider–rail assembly task demonstrated successful sim-to-real transfer, achieving an 82.0% success rate without task-specific fine-tuning.

## 1. Introduction

With the rapid evolution of artificial intelligence, robots are undergoing a profound transformation from conventional automated machinery to embodied intelligent systems capable of perception, reasoning, and decision-making. In unstructured application scenarios such as flexible assembly lines in intelligent manufacturing, domestic services for aging societies, and healthcare, robots are no longer regarded merely as mechanical devices executing pre-programmed instructions. Instead, they are expected to interact seamlessly with humans via natural language and autonomously complete complex long-horizon tasks. This development trend has driven the deep interdisciplinary integration of computer vision, natural language processing, robot control and other disciplines. Consequently, developing intelligent agents that can comprehend abstract semantic concepts and ground them in the real physical world has become a shared goal pursued by academia and industry [1,2,3,4,5]. This natural-language-driven interaction paradigm not only drastically lowers the barriers to robot deployment and operation, but also enables robots to leverage massive image-text knowledge bases available online to cope with unseen tasks in open-world settings, delivering tremendous socioeconomic value and broad application prospects [6,7,8,9].

In recent years, breakthrough advances in deep learning have fueled vigorous progress in embodied intelligence research. In particular, the proposal of the Transformer architecture has unified the paradigm of sequence modeling and laid the foundation for modern large models [10]. Building on this, Vision-Language Models (VLMs), represented by LLaVA and multimodal large models, demonstrated outstanding capabilities in image-text comprehension and logical reasoning [7,8,9,11,12,13,14,15,16,17,18,19]. Pre-trained on massive cross-modal datasets, these foundation models not only acquire abundant world knowledge but also incorporate visual perception abilities, making them widely regarded as promising candidates to serve as the “brain” of robots [20]. Relying on visual feedback from the current scene, such models can decompose vague human instructions into structured subtask sequences, thus resolving the problem of “what actions the robot should execute” at the logical level. Nevertheless, despite the immense practical potential of general large models, a series of recent benchmark evaluations and analytical studies have revealed that directly deploying these models for manipulation tasks involving physical contact still poses notable practical challenges [1,7,8,9].

Cognitive bias constitutes one of the practical challenges. At the cognitive planning level, VLMs are essentially probabilistic sequence generators. Despite incorporating visual information, these VLMs still lack domain-specific expertise for individual tasks and struggle to accurately perceive the spatial geometry of scenes as well as the real-time states of objects. For instance, while VLMs can identify workpieces, they generally have no knowledge of process sequences for industrial assembly or fail to precisely judge geometric collision constraints of tools in narrow spaces. As a result, when tackling long-horizon tasks, these models tend to generate defective planning outputs that comply with semantic logic yet violate operational norms or spatial geometric constraints. Moreover, they struggle to respond quickly to dynamic environmental changes.

Despite extensive prior research along these directions, two critical gaps remain insufficiently addressed. First, existing scene-graph-based planners such as SayPlan [21] and ConceptGraphs [22] focus primarily on spatial geometric representation but lack mechanisms for incorporating temporal manipulation logic from demonstration videos, making them reliant on the VLM’s inherent common sense for task ordering and prone to logical errors in procedurally complex tasks. Second, closed-loop replanning methods including ReplanVLM [13] and DEPS [23] enable failure recovery through visual feedback, but their failure diagnosis operates without reference to standardized manipulation priors, leading to trial-and-error-style replanning that may repeat the same class of errors. Consequently, there lacks a unified framework that jointly exploits temporal logic priors from demonstrations and spatial geometric constraints from scene graphs to improve both planning correctness and execution robustness.

To address the lack of physical geometric constraints and empirical knowledge guidance for robotic manipulation of complex tasks at the cognitive planning level, this work proposes an adaptive task planning framework based on video priors and dynamic scene graphs (ATP-VPDSG). Its core idea is to provide two complementary types of constraints to a Vision-Language Model (VLM). First, video demonstrations are leveraged to extract manipulation prior knowledge that describes the standardized temporal logic of tasks. Second, dynamic scene graphs embedded with visual markers and unique object identifiers (IDs) deliver explicit physical constraints corresponding to the current scene. These two components are integrated into a unified reasoning context for initial planning, execution verification, failure diagnosis and replanning, which effectively improves the autonomous operation capability and intelligence level of robots on complex long-horizon tasks. From a deployment perspective, the modular design of the proposed framework—with decoupled prior extraction, scene representation, and planning modules—also aligns with the service-oriented architecture of industrial Robot-as-a-Service (IRaaS) paradigms, where cognitive capabilities can be delivered as cloud-side services to edge-deployed robots [24].

The main contributions of this work are summarized as follows: (1) A video prior extraction module is proposed to transform video demonstrations into standardized manipulation prior knowledge based on skill primitives and structured prompts. (2) A module of dynamic scene graph based on visual prompting is designed to eliminate ambiguous object references and represent object states and spatial topological relations. (3) An adaptive closed-loop planning mechanism is constructed, incorporating visual expectation verification, rapid logical updates, failure diagnosis, and context reconstruction. (4) The proposed method is evaluated on the LIBERO-10 benchmark with Qwen3-VL as the core VLM, and the results demonstrate that it simultaneously improves planning accuracy and execution success rate compared with selected VLM-based planning baselines [25,26,27].

The remainder of this paper is organized as follows: Section 2 reviews related work; Section 3 describes the design of the proposed method; evaluation experiments, comparative experiments, ablation studies and real-robot experiment are performed to verify the proposed method, and the experimental results are presented and discussed in Section 4; Section 5 concludes this work.

## 2. Related Work

In the embodied intelligence system, which is responsible for transforming abstract instructions into low-level action sequences by integrating scene information, task planning serves as a cognitive bridge connecting semantic intentions and physical control. Its core challenge lies in how to deeply integrate high-level semantics with environmental geometric states, and accurately map them into executable actions that are continuous and comply with physical constraints. To address the logical complexity in long-horizon tasks and the implementation challenges associated with low-level geometric constraints, researchers have conducted extensive investigations.

### 2.1. LLM-Based Planning Paradigms

A recent survey provides a comprehensive taxonomy of LLM-based (LLM, Large Language Model) task planning for service robots, categorizing methods by input modality into text-based, vision-based, audio-based, and multimodal approaches [28]. Within the text-based category, early research primarily focused on integrating large models into the conventional Task and Motion Planning (TAMP) frameworks. To address the lack of logical guarantees in direct planning by large models, LLM+P converted natural language into standard Planning Domain Definition Language (PDDL) descriptions, and leveraged classical solvers to guarantee the causal correctness of actions [29]. To achieve deep integration of semantics and geometry, Auto-TAMP adopted a neuro-symbolic architecture: after the large model generated an action skeleton, it optimized and solved continuous action parameters by combination with signal temporal logic [30]. In addition, to address the state space explosion in large-scale environments, SayPlan constructed a hierarchical mechanism based on three-dimensional (3D) scene graphs, realizing efficient and scalable planning in large-scale spaces through iterative semantic pruning and on-demand loading of local geometric information [21].

Compared with TAMP methods that rely on domain-specific files, the programmatic paradigm based on code generation was gradually emerging as a new trend for expressing complex logical strategies, owing to its strict syntax and control flow. Among these efforts, Code as Policies (CaP) proposed the “code as policy” paradigm, which allowed large models to directly generate Python code containing perception Application Programming Interfaces (APIs) and control flows, thereby enabling the handling of complex logical tasks that depend on real-time states [31]. To address the challenge of environmental perception grounding, ProgPrompt encapsulated available objects and robotic skills into a Pythonized contextual environment, converting the planning process into a code completion task; this approach effectively avoided object reference errors in open-world scenarios [32]. To standardize code logic and improve safety, Vemprala et al. systematically established code generation principles based on “predefined high-level API library calls” [33].

To realize deep alignment between semantic logic and physical affordance, SayCan proposed a probabilistic grounding mechanism [34]. This mechanism calculated a comprehensive score by multiplying the skill semantic probability output from the large model by the physical feasibility evaluated via the reinforcement learning value function, and greedily selected the action with the highest score for execution. This guaranteed that the planning results were physically valid and feasible. Aiming at the problem that Large Language Models (LLMs) lack foresight in long-horizon planning, RAP combined LLMs with Monte Carlo Tree Search (MCTS); during the planning phase, it performed multi-step mental simulations to derive the globally optimal action sequences [35]. To address the problem of LLMs’ interaction with the environment, Luan et al. proposed a multi-layer model that integrated an LLM with a VLM and a calorific heat map [36]. This approach facilitated a multi-layer decomposition of tasks, thereby elevating the precision of task decomposition and enhancing the LLM’s proficiency in comprehending intricate real-world tasks.

### 2.2. Visual Grounding and Scene Representation

With the rapid development of VLMs, direct vision-language planning has become an increasingly prominent direction, aiming to eliminate the reliance on external detectors and realize end-to-end alignment between raw visual information and task planning. VILA proposed a direct vision-language planning architecture that leveraged visual chain-of-thought (CoT) to guide the VLM to first analyze the scene layout and then generate action sequences, effectively addressing complex multimodal tasks [11]. Building on this foundation, ViLaIn constructed a symbolic correction closed loop that converted perception results into PDDL descriptions; when the classical planner failed to find a solution, the error information was used to backward guide the large model to re-examine the images, thereby dynamically correcting misjudgments of object relations [12]. More recently, advances in multimodal foundation models have further pushed the frontier of VLM-guided planning, with unified architectures and enhanced visual prompting capabilities demonstrating significant improvements in perception-action alignment for robotic manipulation.

A closely related line of research focuses on structured scene representations to improve spatial reasoning and reduce perceptual hallucinations. ConceptGraphs extracted object features using Segment Anything Model (SAM) and Contrastive Language-Image Pre-training (CLIP), fused them into 3D point clouds, and constructed an open-vocabulary-based 3D scene graph that enables semantic search and reasoning directly in 3D space [22]. Prompt2Act innovatively integrated semantic visual augmentation and a dynamic hybrid execution strategy to address ambiguous fine-grained localization and monotonous execution policies, realizing accurate and efficient mapping from multimodal prompts to robotic action sequences [14]. Statler introduced a world state machine mechanism that leverages the LLM to explicitly maintain an environmental state graph and updates node states in real time after each action, endowing the robot with long-term and stable state memory capabilities [37].

### 2.3. Closed-Loop Replanning and Failure Recovery

To address the problem that open-loop planning in real physical environments is prone to failure due to execution errors, constructing a closed-loop system with failure diagnosis and self-reflection capabilities has become a research frontier. ReplanVLM proposed a two-layer feedback mechanism for perception and cognition [13]. At the low level, this method corrected grasping poses in real time through visual feature matching, while at the high level, it triggered the VLM to regenerate code logic when the execution was blocked by environmental changes. To further tackle the challenge of causal attribution for planning failures, DEPS proposed a closed-loop mechanism consisting of “Description-Explanation-Planning-Selection” [23]. When execution failed, this mechanism guided the VLM to first generate a failure explanation based on current observations, and then formulate a targeted revision plan accordingly, thereby enabling robust planning with human-like reflection capabilities. To improve the logical correctness and stability of long-horizon tasks, ISR-LLM proposed an iterative self-improvement framework combined with PDDL formal representation [38]. This method constructed a closed-loop mechanism consisting of translation, generation, verification, and revision. It leveraged a logical verifier to generate feedback, guiding the large model to continuously revise action sequences until logical consistency was achieved, which significantly reduced the logical error rate.

Despite promising progress in the aforementioned research, two critical gaps remain insufficiently addressed when coping with complex unstructured scenes. First, existing scene graph and spatial representation methods such as SayPlan [21], ConceptGraphs [22], and Statler [37] focus primarily on geometric state representation but lack mechanisms for incorporating temporal manipulation logic from video demonstrations, leaving task ordering entirely to the model’s zero-shot commonsense reasoning. Second, existing closed-loop replanning methods including ReplanVLM [13] and DEPS [23] enable failure recovery through visual feedback, but their failure diagnosis operates without reference to standardized manipulation procedures, leading to trial-and-error-style recovery that may repeat the same error class. Prompt2Act [14] introduces visual prompting for improved grounding but does not construct explicit topological scene graphs nor integrate video demonstration priors for temporal logic guidance. Consequently, there lacks a unified framework that jointly exploits video-derived temporal logic priors and dynamic scene-graph-based spatial constraints within a closed-loop replanning pipeline. This work addresses this gap by integrating these two complementary constraints into a dual-track closed-loop planning mechanism, improving both the accuracy of initial planning and the robustness of execution recovery.

## 3. Methods

### 3.1. Overview of ATP-VPDSG

**Figure 1:**
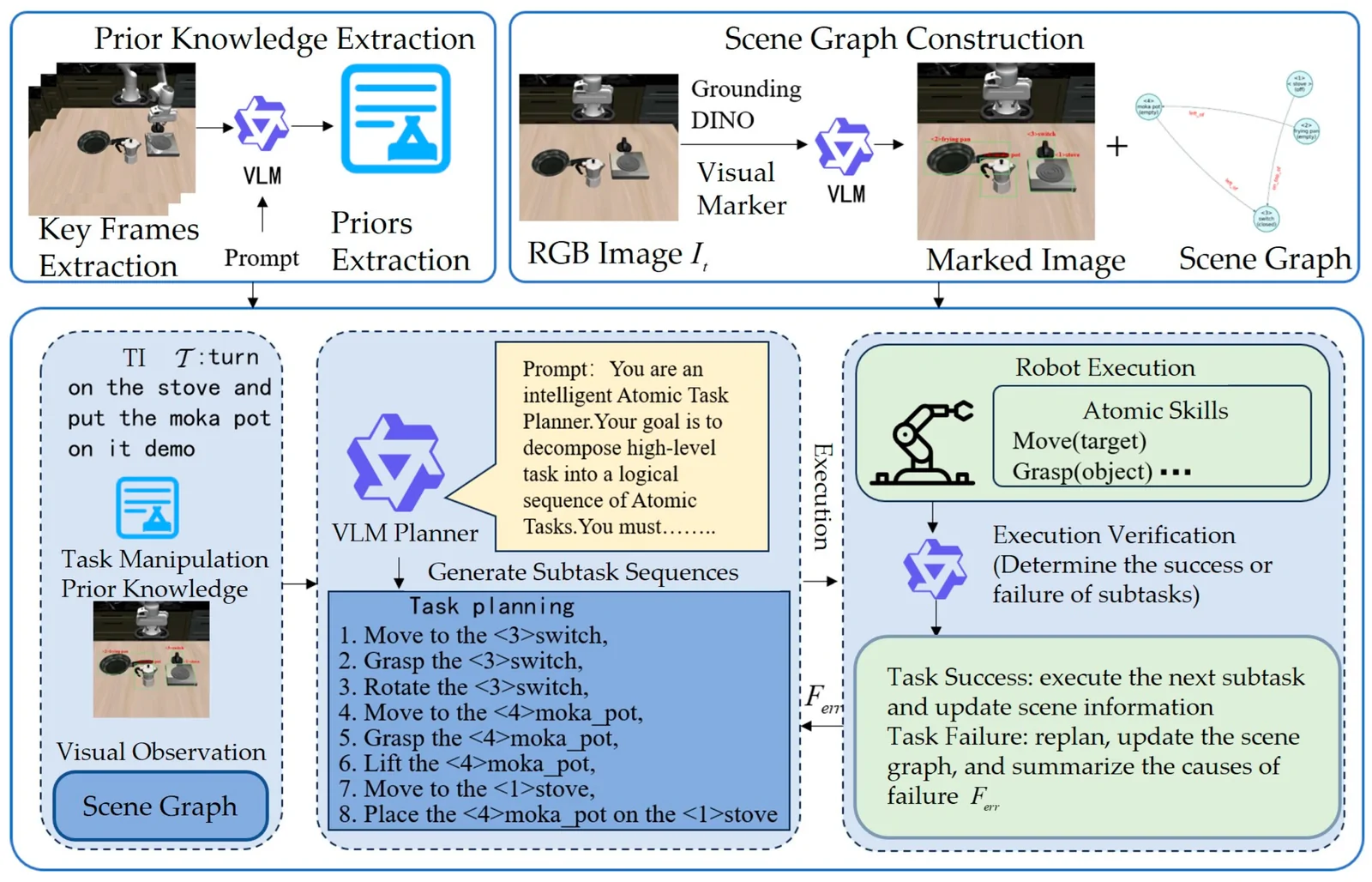
Overall framework of ATP-VPDSG.

This section presents the detailed design of ATP-VPDSG. As illustrated in Figure 1, the framework leverages the semantic understanding capability of the VLM to integrate prior knowledge extracted from video demonstrations and real-time environmental observations into a unified reasoning context, thereby generating executable manipulation sequences. Its architecture consists of three core modules as elaborated below.

1. Video Prior Extraction: This module leverages the VLM to extract standardized task logic skeletons from video demonstrations. By analyzing key states within videos, it converts implicit manipulation experiences into temporally dependent task logic priors, which provides high-level behavioral guidance for planning;
2. Dynamic Scene Graph Construction: This module adopts visual prompting strategy based on the VLM to convert unstructured environmental visual information into symbolic constraints. It further identifies object attributes and spatial topological relations to construct a real-time updatable scene graph, providing the planner with explicit physical entity indexes and geometric constraints;
3. Adaptive Task Planning: Guided jointly by logic priors and geometric constraints, this module generates concrete manipulation sequences. Meanwhile, it incorporates a closed-loop feedback mechanism that receives state feedback during execution, analyzes the causes of execution failures, and performs dynamic replanning to guarantee successful task completion.

### 3.2. Problem Definition

Task planning for robotic manipulation in complex scenarios can be fundamentally formulated as a sequential decision-making problem that maps abstract task instructions to physical-space state transitions. Formally, given abstract task instructions T and environmental observations $I_{t}$, the core objective is to construct a decision mapping that decomposes the high-level abstract instructions into finite-length ordered subtask sequences $S=\{a_{1},a_{2},\ldots ,a_{n}\}$. By executing these subtasks in sequence, the robot drives the environmental state to transition toward the target state satisfying the expectations specified by the instructions.

To guarantee executability and robustness of the sequences in real physical environments, this decision mapping must satisfy two core constraints. The first is the logical and spatial constraint: the sequences should conform to the causal manipulation logic of the task and align with the geometric topological state of the current scene. The second is closed-loop planning: task progress is verified by comparing environmental observations before and after subtask execution, enabling timely intervention and replanning in cases of environmental disturbances or execution failures.

### 3.3. Extraction of Manipulation Priors from Video Demonstrations

Compared with simple language instructions, video demonstrations contain substantially richer environmental interaction details, while also providing stronger semantic abstraction than trajectory data. To transform such unstructured visual streams into standardized manipulation procedures executable by robots, this work proposed a VLM-based semantic parsing method. The method first performs state compression on raw videos, and then leverages structured prompts to guide the model in mapping visual observations to predefined atomic manipulation sequences.

For the collected video demonstrations, the data can be formally represented as a temporally continuous observation sequence $V_{\mathrm{ori}}=\{I_{1},I_{2},\ldots ,I_{T}\}$. However, the raw sequences contain substantial redundancy (e.g., operation pauses, smooth motion transitions, etc.). To construct informative key frames tailored for reasoning with the VLM, the proposed method adopts an adaptive sampling strategy based on structural similarity. This algorithm captures abrupt state change moments in the scene by monitoring inter-frame visual differences via $D(t)=1-\mathrm{SSIM}(I_{t},I_{t-1})$. Rather than performing uniform sampling, the algorithm is grounded in vision–physics correspondence assumption, namely that key physical interactions in the real world (e.g., significant motion, grasping, occlusion changes, etc.) inevitably induce significant sudden mutations of structural features in the image plane. By setting an adaptive threshold and introducing a local peak preservation mechanism, the system can automatically skip meaningless smooth transitions while accurately pinpoint the instants of qualitative physical state shifts. This SSIM-based sampling strategy inherently exhibits robustness against common interference in demonstration videos. Since structural similarity captures global structural feature changes rather than pixel-level intensity fluctuations, it is insensitive to moderate Gaussian noise and illumination variations. The adaptive threshold mechanism dynamically adjusts detection sensitivity according to the overall variation amplitude of the video, ensuring stable keyframe detection under low-frame-rate inputs. For partial occlusion during manipulation, the local peak preservation mechanism retains frames corresponding to contact-induced structural mutations, avoiding missed detection of critical state transitions caused by transient occlusion. Consequently, the raw videos are compressed into a compact discrete state sequence $V_{key}=\{I_{1},I_{2},\ldots ,I_{N}\}$, where N<T. Each frame in the sequence represents a “key state slice”, while the temporal information of actions is preserved across consecutive frames, providing critical cues for downstream semantic reasoning.

To bridge the semantic ambiguity of the VLM in action comprehension (e.g., describing a grasping action as “holding” or “taking”, etc.), a set of standardized basic skill primitives is defined. By constraining the outputs of the VLM, semantic ambiguity is eliminated and the generated instructions are guaranteed to be physically executable. Considering the task scenarios, this work categorizes the output actions into four major types of basic skill primitives, as listed in Table 1, so as to constrain the VLM to extract standardized manipulation prior knowledge and output structured executable instructions.

The defined primitive set is sufficient to cover all manipulation patterns in the LIBERO-10 benchmark, including spatial navigation, pick-and-place, articulated object interaction, and part alignment. For broader application domains, the primitive set supports hierarchical extension at three levels:

Parameter-level extension: Existing primitives (e.g., “Rotate”, “Push”) can be parameterized with additional attributes such as angle range and force profile to accommodate finer-grained control requirements without introducing new primitive types.

Primitive-level extension within categories: New primitives (e.g., “Screw”, “Pour”) can be added under the four existing categories by extending the prompt template with few-shot exemplars and updating the low-level skill mapping interface. This extension is straightforward, as the core reasoning pipeline remains unchanged and no VLM fine-tuning is needed.

Category-level extension: For substantially different domains (e.g., mobile manipulation), entirely new primitive categories can be introduced. The overall video prior extraction pipeline (keyframe sampling + CoT reasoning + structured output) remains architecturally unchanged; only the primitive library and corresponding exemplars require expansion.

This design ensures that the extraction framework is domain-agnostic at the architectural level, with the primitive set serving as a pluggable interface layer.

After defining standardized set of basic skill primitives, the key challenge is how to enable the VLM to infer normalized manipulation sequences that are both physically plausible and semantically constrained from static discrete key frames. Conventional question-answering prompts are generally failed to prevent the model from generating either factually inaccurate or non-standardized manipulation sequences. To fully exploit the potential of the VLM in comprehension of unstructured video frames and to transform the comprehension of video demonstrations into reasonable robotic manipulation sequences, this work proposed a structured system prompts design based on CoT reasoning and in-context learning. The prompt framework consists of three main components: expert role and task-context definition, CoT reasoning guidance with exemplar-based in-context learning, action semantic standardization and manipulation knowledge encapsulation. The prompts explicitly define the model’s role and objectives, embeds structured reasoning logic, and constrains output formats, thereby prompting the model to act from the perspective of a manipulation parsing expert and conduct in-depth parsing of manipulation sequences in frames of video demonstrations.

**Figure 2:**
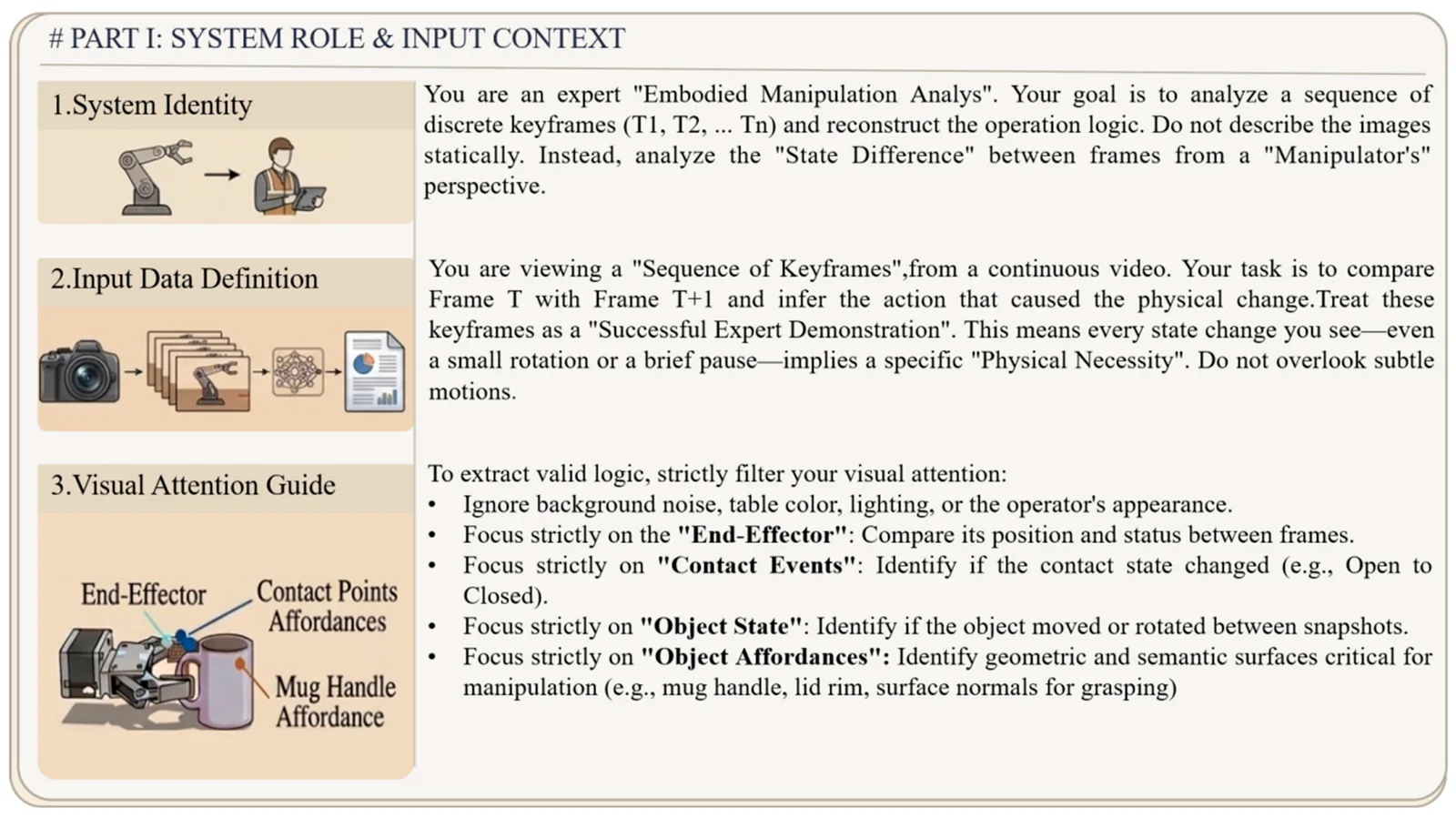
Prompt design for role definition and task-context grounding.

At the initial stage of analyzing and extracting manipulation prior knowledge from video demonstrations, it is essential to establish the role and tasks of the VLM. Accordingly, the first component of the prompt framework is designed to realize cognitive perspective shift and task-context grounding. In this component, the model is explicitly defined as an “embodied manipulation analysis expert”, whose responsibility is to summarize and extract manipulation knowledge from given expert demonstrations. The prompt design for role definition and task-context grounding is illustrated in Figure 2. Furthermore, the prompt specifies that the model should not merely describe individual images in isolation. Instead, it is required to infer motion states of robotic arms or human hands by analyzing state-difference across several adjacent static frames. For example, if the hand is resting on the table in the previous frame and appears suspended above the table in the subsequent frame, the VLM is expected to infer a lifting action based on physical commonsense reasoning, rather than simply describing positional changes.

In addition, to prevent the model from overlooking subtle manipulation details when processing long manipulation sequences, the prompt explicitly instructs the model to attend to every action in the expert demonstrations, thereby guaranteeing the completeness of the extracted manipulation prior knowledge. Meanwhile, to mitigate the interference of background noise, the prompt also establishes a strict visual attention checklist, which requires the model to ignore background textures, lighting conditions and the appearance of the operator, and concentrating computational resources intensively on three core elements: end-effector trajectories, object contact states, and geometric configuration variations.

**Figure 3:**
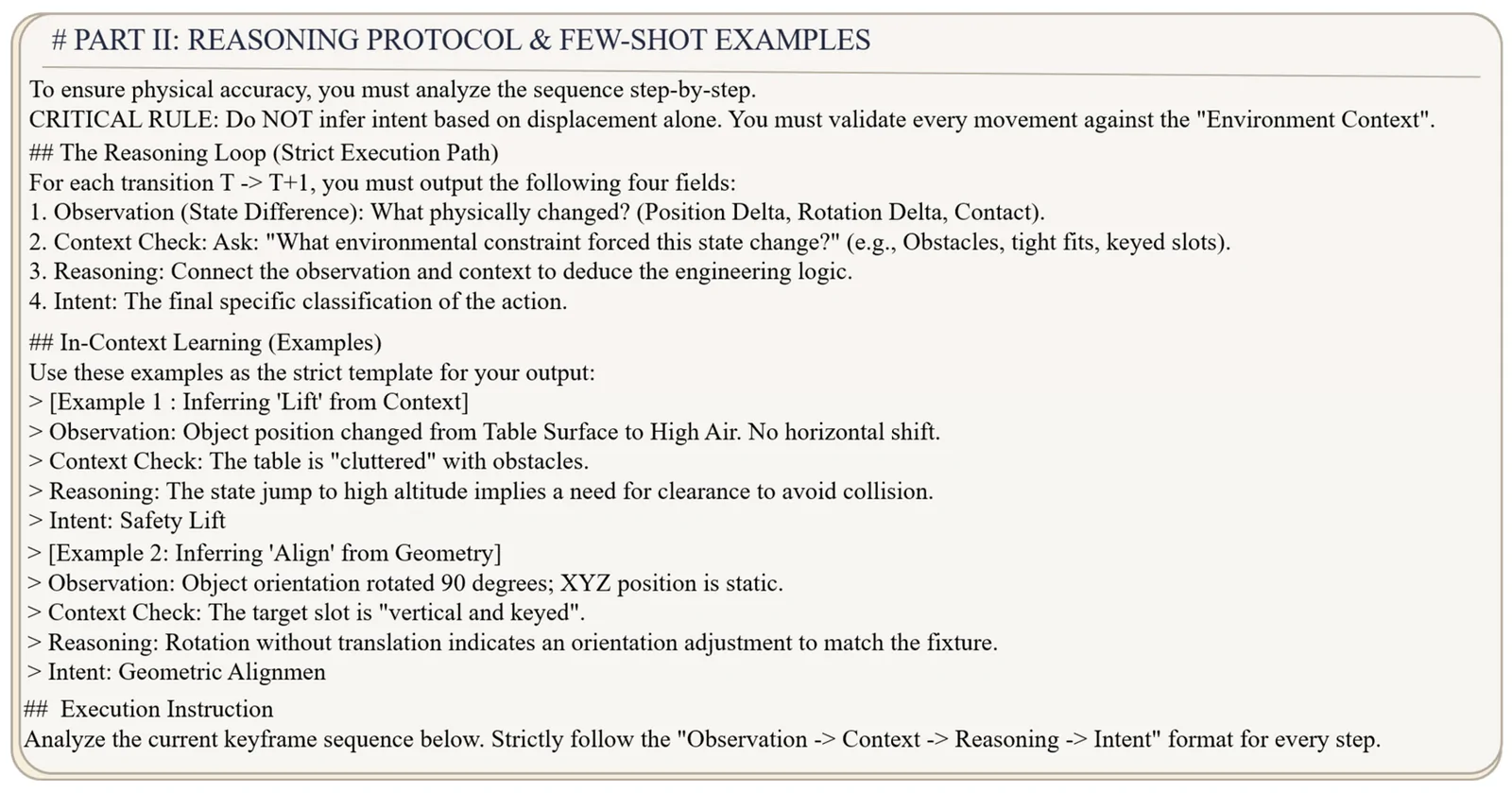
Prompt design for manipulation intention reasoning based on CoT and few-shot exemplars.

After establishing the observation paradigm of inferring actions from state differentials, the central challenge lies in deriving the correct physical intention from visual observations. Due to temporal gaps between key frames, motion trajectories are inherently incomplete, and an identical trajectory may correspond to entirely different manipulation intents under different environmental contexts. For instance, a vertical displacement of an object may indicate either the initiation of a routine pick-and-place operation or collision avoidance motion. If the model judges solely based on geometric displacement information, it is highly prone to logical fallacies. To address this issue, the second component of the proposed prompt framework constructs a deep reasoning protocol built upon CoT, combined with few-shot exemplars to guide the model in inferring plausible manipulation intents by leveraging environmental context, as illustrated in Figure 3. This reasoning protocol mandates the model to complete a full thinking loop consisting of state-difference observation, contextual attribution and intention confirmation, prior to output final conclusions.

First, the model is required to objectively record the state variations between consecutive frames, including shifts in end-effector positions, rotational posture offsets, and alterations in object contact states. Afterwards, the prompt guides the model to actively retrieve environmental features for causal attribution. The model is instructed to raise self-reflective questions, such as “Why does the height change sharply at this step? Is it caused by surrounding obstacles?” or “Why does an in-place rotation occur? Does the target slot feature a special shape?” This mechanism compels the model to leverage environmental constraints to infer missing information gaps between key frames. To enable the model to truly master this reasoning capability, expert exemplars are directly embedded within the prompts. These examples demonstrate complete thinking chains that deduce lifting an object from two seemingly simple static–motion snapshots with the gripper holding the object amid cluttered environment, as well as deducing alignment operations by referencing irregular-shaped slots. By imitating these exemplars, the model learns to seek supporting evidence from environmental contexts, thus realizing accurate reasoning manipulation intentions.

**Figure 4:**
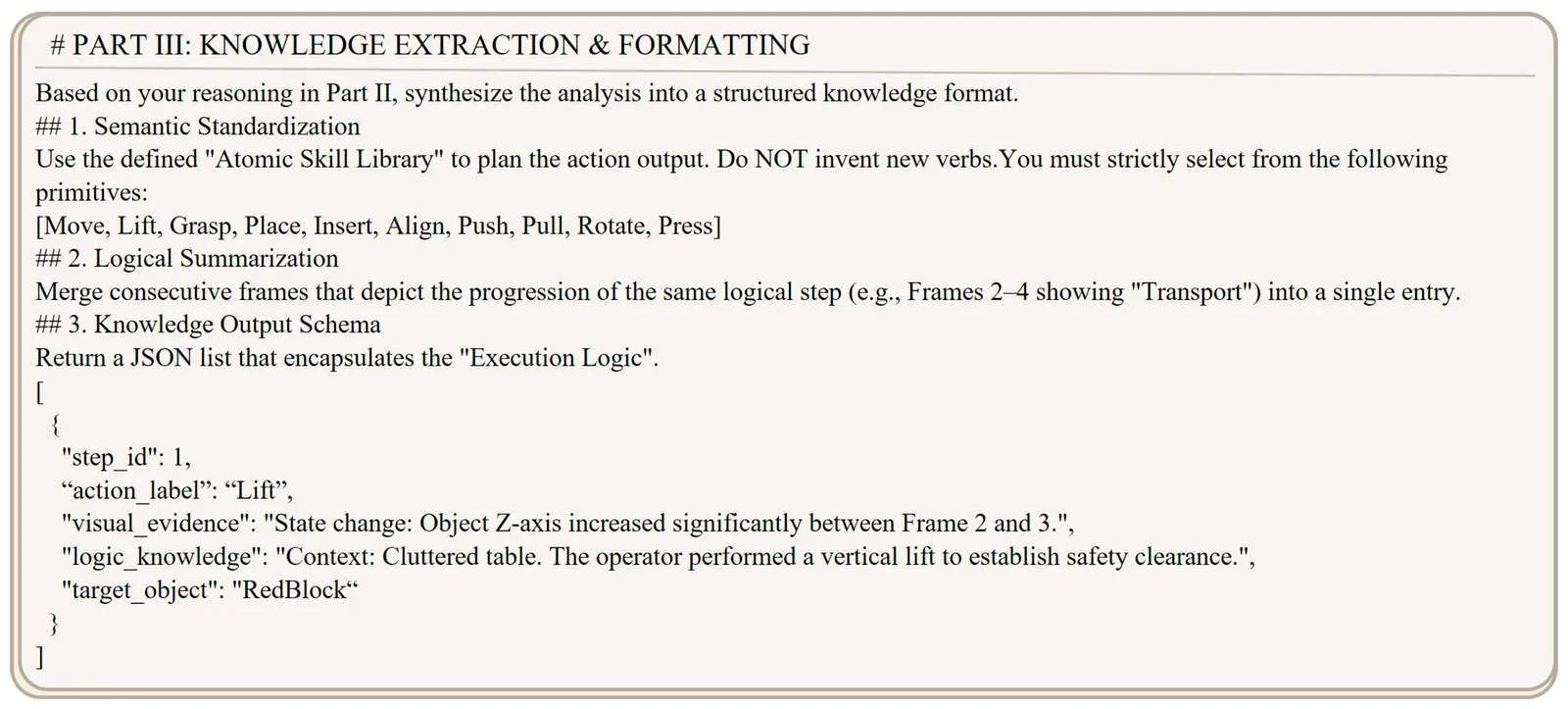
Prompt design for output format definition.

Since the inputs are discrete key frames, the directly generated natural language descriptions may suffer from temporal incoherence or arbitrary semantics (e.g., mixed use of grasping and holding). To convert the implicit manipulation knowledge contained in video demonstrations into explicit structured knowledge, the final component of the prompt framework focuses on standardizing manipulation sequences via predefined skill primitives and realizing structured encapsulation of manipulation knowledge, as illustrated in Figure 4. First, the prompt compels the model to select manipulation exclusively from the predefined basic skill library, thereby guaranteeing that high-level intentions derived via CoT reasoning are accurately mapped to a closed set of standardized verbs. Second, the prompt performs manipulation knowledge encapsulation. To tackle temporal redundancy in keyframe sequences, the prompt mandates the model to perform logical merging, aggregating consecutive frames representing identical logical steps into a single knowledge entry. Finally, the system enforces outputs in JSON format with mandatory logical knowledge fields that fully record environmental constraints and decision-making evidence. This design not only records the manipulations performed by the expert but also preserves the environmental conditions underlying each decision, providing high-quality prior knowledge for downstream robotic task planning.

While the four primitive categories defined in Table 1 are sufficient to cover the manipulation patterns in the LIBERO-10 benchmark, the primitive set is designed to be extensible for broader application domains. The core extraction pipeline—adaptive keyframe sampling, CoT-structured prompting, and JSON-formatted output—is domain-agnostic at the architectural level, with the primitive set serving as a pluggable interface layer between general VLM reasoning and domain-specific execution. For new task domains such as industrial assembly, additional primitives (e.g., insert, screw, press) can be introduced under the existing state-changing category by extending the prompt template with new few-shot exemplars, requiring only prompt-level modifications without changing the pipeline. For substantially different domains such as mobile manipulation or tool-use tasks, entirely new primitive categories can be added while the extraction pipeline remains unchanged.

### 3.4. Construction of Scene Graphs Based on Visual Prompting

Although VLMs exhibit remarkable semantic generalization capabilities on general image understanding tasks, directly transferring these abilities to robotic task planning still suffer from a critical perception–reasoning misalignment challenge. Raw RGB images contain abundant environmental information yet inherently lack the explicit spatial structure and deterministic object references required by planners. In unstructured complex environments, VLMs often fail to disambiguate specific manipulation instances among multiple objects of the same category (e.g., failing to distinguish the left red bowl and the right red bowl). Meanwhile, they cannot directly parse relative spatial relations between objects from pixel data, such as occlusion, containment, and relative orientation. Such drawbacks of ambiguous references and vague spatial relations readily cause the planner to generate semantically feasible yet physically implausible instructions. To address this issue, this work adopted an active visual prompting strategy. By overlaying visualizable identifier (ID) labels and semantic labels onto the raw images, this strategy actively guides the VLM to focus its attention on critical physical instances. This processing pipeline converts ambiguous continuous visual signals into clear discrete symbolic indices, thereby providing a set of accurately addressable structured symbolic representations free of referential ambiguity for complex long-horizon task planning.

The dynamic scene graph construction pipeline consists of three functionally complementary sub-components, each addressing a distinct perception-reasoning misalignment problem:

- Grounding DINO-based open-vocabulary detection: This sub-component performs bottom-up instance perception from raw RGB images. Its core value lies in enabling zero-shot detection of novel object categories without retraining, laying the foundation for open-world deployment. Without this component, the system would be restricted to object categories recognizable by the VLM’s endogenous visual perception.
- Unique ID visual annotation: This sub-component solves the referential ambiguity problem by assigning a globally unique numerical ID to each detected instance and overlaying it on the image. It eliminates the “which one” problem in multi-object scenes with semantically similar items, which is a dominant source of planning errors. This is achieved purely through visual prompting without additional model training.
- Topological spatial relation parsing: This sub-component extracts relative spatial relations (left/right, inside/on-top, front/behind) between objects and organizes them into a graph structure. It provides explicit spatial reasoning primitives, reducing the VLM’s burden of inferring geometric relations from raw pixels and mitigating spatial hallucinations.

The three sub-components operate sequentially in a pipeline: detection provides the complete instance set, ID annotation establishes unambiguous referenceability, and relation parsing constructs the structured spatial representation for planning.

**Figure 5:**
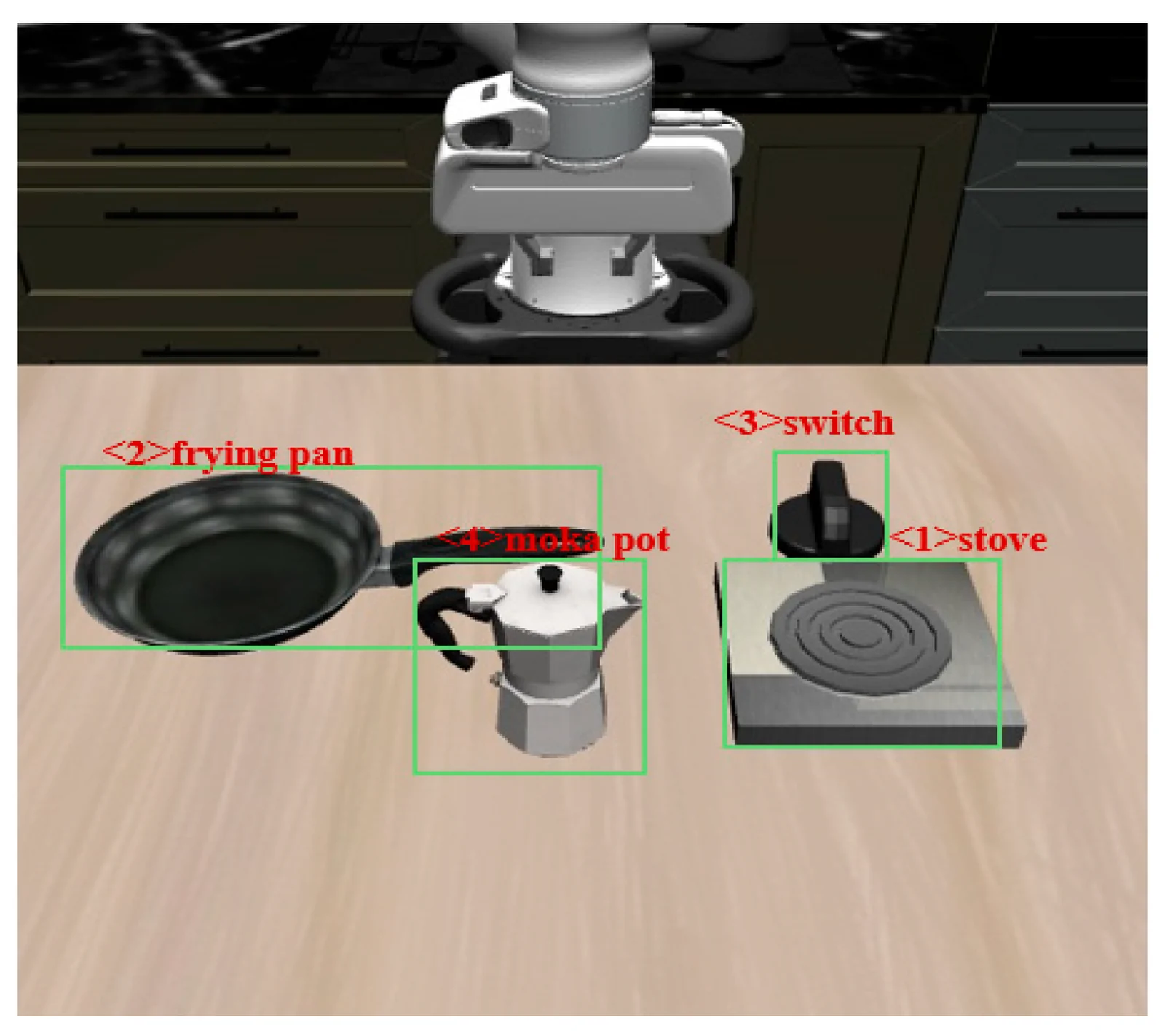
Annotated image with assigned IDs for visual prompts.

The first step of the scene graph construction proposed in this work establishes alignment between visual space and the symbolic space to fundamentally resolve the problem of referential ambiguity in multi-object scenes. In scenes with multiple objects, relying merely on natural language descriptions often fails to distinguish visually similar objects, which makes the abstract instructions output by the planner unable to target specific physical entities accurately. To tackle this challenge, this work draws on the Set-of-Mark (SoM) visual prompting paradigm to construct a rigid “what-you-see-is-what-you-reference” mapping. First, the proposed method leverages the VLM to prompt Grounded-DINO to perform open-vocabulary detection on the RGB observation image of the current task scene, segmenting the visual regions of interactive objects from complex backgrounds [39]. To address this issue, the proposed method adopts the visual prompting paradigm of Set-of-Mark (SoM) to establish a strict “what-you-see-is-what-you-refer-to” mapping. Subsequently, each extracted physical instance is assigned a globally unique numerical ID. High-contrast labels consisting of IDs and semantic categories are overlaid precisely along the object contours of the original image, producing a marked image $I_{mark}$. This pipeline of detection, bounding-box annotation and re-input encapsulates the originally continuous and indistinguishable pixel regions into discrete symbols. For the downstream VLM-based planner, image processed with visual prompts is no longer a pure visual signal but a numbered inventory of object, as illustrated in Figure 5. This processing endows the VLM with a handle mechanism analogous to that in programming languages, enabling it to precisely reference specific objects via unique IDs and fundamentally eliminate referential ambiguity inherent in natural language.

After confirming the identity of each entity, the system further performs semantic abstraction on the spatial positions of entities to support logical reasoning of the planner. Unlike conventional approaches that directly record absolute coordinate information, the proposed method aims to extract the relative spatial layouts among objects. Leveraging the outstanding commonsense reasoning capability on images of the VLM, this system parses relative spatial orientations and containment relations between nodes combined with visual markers. Guided by predefined prompts, the model conducts comprehensive inference based on marked pixel coordinates and visual features within labeled regions. Specifically, the VLM derives relative object positions from the relative distribution of markers and image comprehension, such as left/right and front/behind. Furthermore, relying on marker overlap and semantic understanding, the VLM is able to infer more complex spatial relations. For example, when the annotated bounding box of object A lies within the contour of object B, the VLM combines commonsense semantics to judge pairwise spatial relations as inside (A, B) or A on top of B. This method fully exploits the VLM’s capability to comprehend complex visual relations while incorporating the explicit unique indices provided by visual markers, thereby enabling concise and accurate representation of real-world environmental layouts.

**Figure 6:**
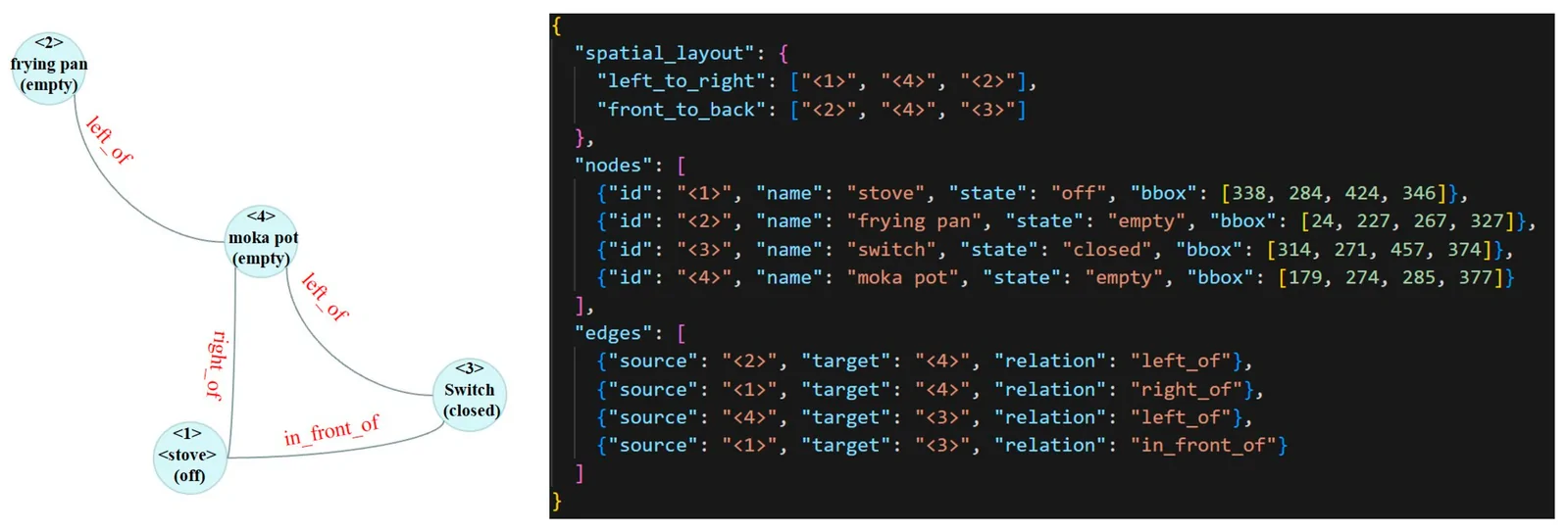
JSON format of the scene graph (**right**) and its corresponding visualized topological graph (**left**).

To support complex reasoning for subsequent task planning, this work formalizes the aforementioned perceptual information into a dynamically updated semantic scene graph $G_{t}$. Within the graph structure, each node in the node set stores an assigned unique logical ID, semantic category label, and object state. Meanwhile, the edge set records the topological spatial relations between objects and additionally maintains a global spatial sorting list, such as object sequences ordered left-to-right and front-to-back. Figure 6 presents a standard JSON instance of the scene graph generated by the system alongside its corresponding visualized topological graph. This structure clearly depicts the physical layout of the task scene: object <4> moka pot is to the left of <2> frying pan, while <2> frying pan lies to the right of <1> stove, and so on.

Such structured scene graph representation supplies VLM-based planner with logically coherent and unambiguous symbolic environment descriptions. It refines and converts the unstructured visual information contained in raw images into logical data consisting of deterministic object indices and spatial topological relations. By introducing explicit symbolic constraints, this scene representation can effectively suppress referential confusion and spatial perception hallucinations induced by scattered visual attention of the VLM in complex scenarios, thus providing accurate and reliable environmental perceptual constraints for task planning.

### 3.5. Adaptive Closed-Loop Task Planning

**Figure 7:**
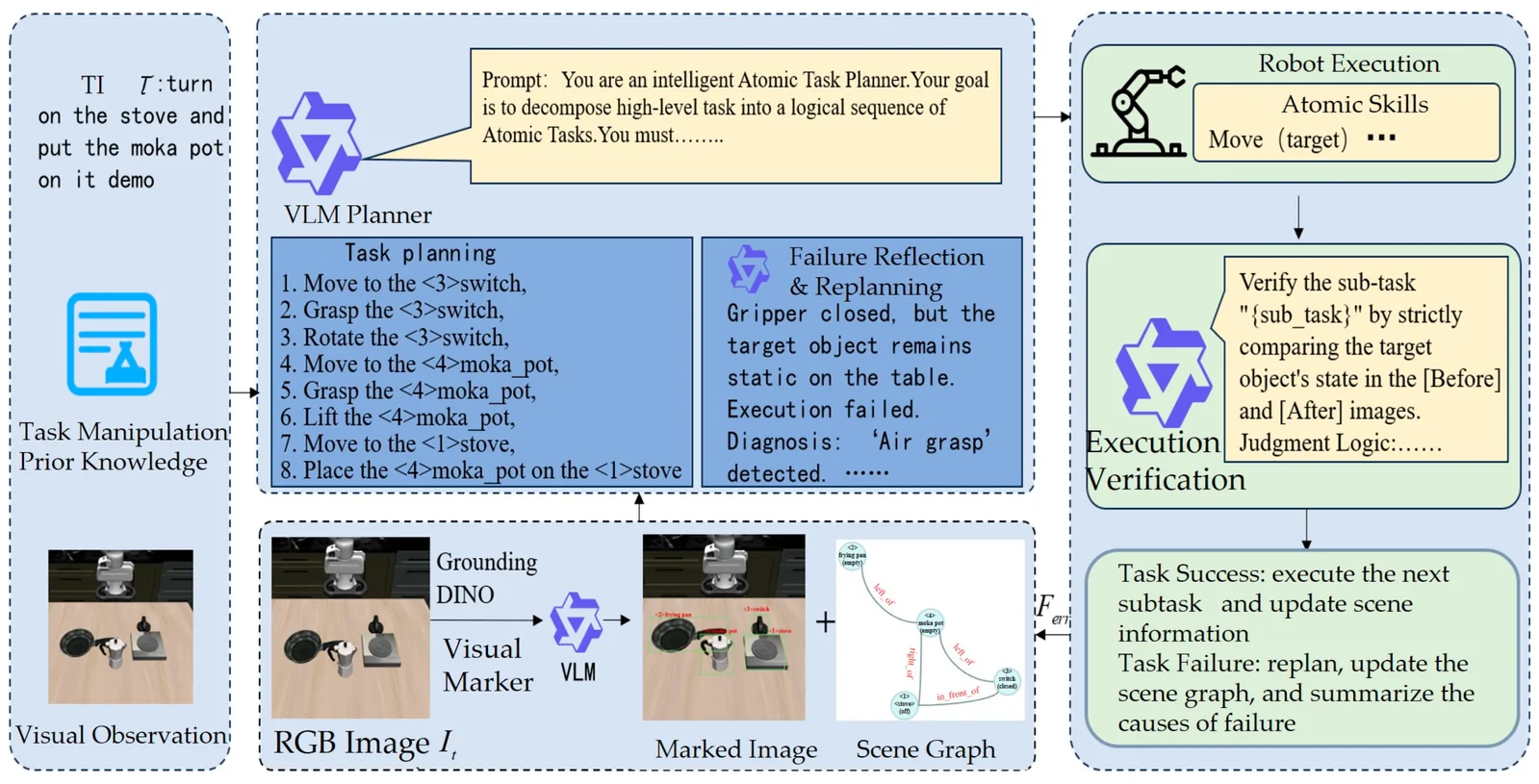
Framework of adaptive closed-loop planning method.

To fully leverage the manipulation prior knowledge extracted in the previous sections as well as the constructed scene graph, this work proposed an adaptive closed-loop planning method. Its framework is illustrated in Figure 7.

The system fuses multimodal information into a unified reasoning context to drive the VLM to generate initial manipulation sequences, and cooperates with a visual verification mechanism to realize closed-loop execution. The detailed procedure of the proposed planning method is presented in Algorithm 1.

At the onset of planning, the system first parses the semantics of the task instructions T and extract instruction features. It then retrieves the best-matched prior execution sequences $K_{prior}$ from the manipulation skill knowledge base built upon manipulation priors extracted from video demonstrations. Such manipulation sequences summarize general experience for completing corresponding types of tasks (e.g., the drawer-opening task: move beside the drawer handle, grasp the handle, pull the drawer open). Nevertheless, these priors essentially consist of generalized semantic descriptions, whose manipulation targets are generic category-level nouns and thus cannot directly guide physical manipulation of individual instance-level objects. To transform this “general reference plan” tailored for a certain task into concrete feasible execution strategy for the current scene, the system fuses visual features, semantic information and spatial structures to form a unified reasoning context C. Within this context, semantic information is composed of textual descriptions of standardized manipulation procedures from manipulation priors and the corresponding names of manipulated objects, which define the specific concepts of tasks. Spatial structure consists of topological relations and node IDs of the scene graph, which serve as indices for physical entities. By leveraging the in-context learning capability of the VLM, the system realizes semantic anchoring to map object nouns contained in prior knowledge to specific object instances in the scene graph. Specifically, the VLM performs an in-depth comparison between generalized concepts extracted from prior knowledge and individual nodes in the scene graph. Relying on the structured indices supplied by the scene graph, the VLM can refer to general manipulation workflows while incorporating visual features of image $I_{t}$, so as to pinpoint broad manipulation targets to concrete node instances within the current environment (e.g., ID: 5, top drawer handle). Essentially, this process instantiates generic manipulation experience into manipulation intentions tailored to specific scenes, effectively bridging the gap in scene adaptation for general manipulation priors.
**Algorithm 1.** Adaptive closed-loop task-planning algorithm**Input:** Task instruction T, Raw observation $I_{0}$, Prior knowledge base $K_{prior}$, Scene-Graph $G_{t}$
**Output:** Task Completion Status1: C ← BuildUnifiedContext(T,$I_{0}$,$K_{prior}$,$G_{t}$)//1. Initialization: Construct a unified reasoning context2: **while** not Task_Completed do//2. Initial Planning: The VLM directly generates task sequences based on the constructed context3:   $A_{t}$ ← VLM(C)4:   **for** each $a_{t}$ in $A_{t}$ do//3. Execution and verification5:     $I_{pre}$,$I_{post}$ ← Execute($a_{t}$)6:    **if** Verify($I_{pre}$,$I_{post}$) is True then//Subtask executed successful, rapid logical 7:       $G_{t+1}\leftarrow {\mathrm{Update}}_{logic}$($G_{t}$,$a_{t}$) 8:    **else**//Subtask execution failed, scene graph update, failure causes analysis, and replan9:      $F_{err}$ ← VLM($I_{pre}$,$I_{post}$)10:     $G_{t+1}$ ← Perception($I_{post}$)11:     C ← ReconstructContext(T,$I_{post}$,$F_{err}$,$G_{t+1}$)12:      **break**//Interrupt execution and trigger replanning 13:   end if14:    end for15:    if AllSuccess ($A_{t}$) then **return** Success end if16: end while17: **return** Failure

In dynamic environments, geometric constraints such as visual occlusion and spatial interference are often implicit and cannot be fully represented explicitly in the scene graph. Mechanically executing generic prior sequences extracted from video demonstrations is easily lead to task failures due to neglect of the unique characteristics of the specific environment. During the planning phase of the proposed method, the scene graph acts not as a rigid rule base but as a structured index to guide the VLM’s visual attention. By adopting CoT techniques, the model focuses on regions of interest in image $I_{t}$ according to anchored node ID information and conducts in-depth analysis of geometric features along manipulation paths. Suppose the prior knowledge defines a scheduled manipulation $k_{i}$ to be executed. Guided by the topological relations of the scene graph, the VLM performs visual analysis and discovers another object $v_{obs}$ near the manipulated object that causes occlusion. Pre-trained on massive internet-scale data, the VLM possesses abundant physical common sense. During CoT reasoning, it can infer that directly executing the predefined manipulation $k_{i}$ will trigger a collision between the robotic manipulator and the occluding object $v_{obs}$, resulting in task failure. Through this CoT-guided causal reasoning, the VLM autonomously revises the manipulation sequence derived from general priors and generates a new task plan to guarantee smooth task execution. For instance, it dynamically inserts the subtask “remove the occluding object” into the original sequence. This mechanism leverages the generalized reasoning capabilities of the VLM to compensate for the blindness and limitations of static prior knowledge when coping with dynamic environmental changes. It guarantees that each subtask not only conforms to task logic specifications but also strictly satisfies the feasibility constraints of the physical world.

**Figure 8:**
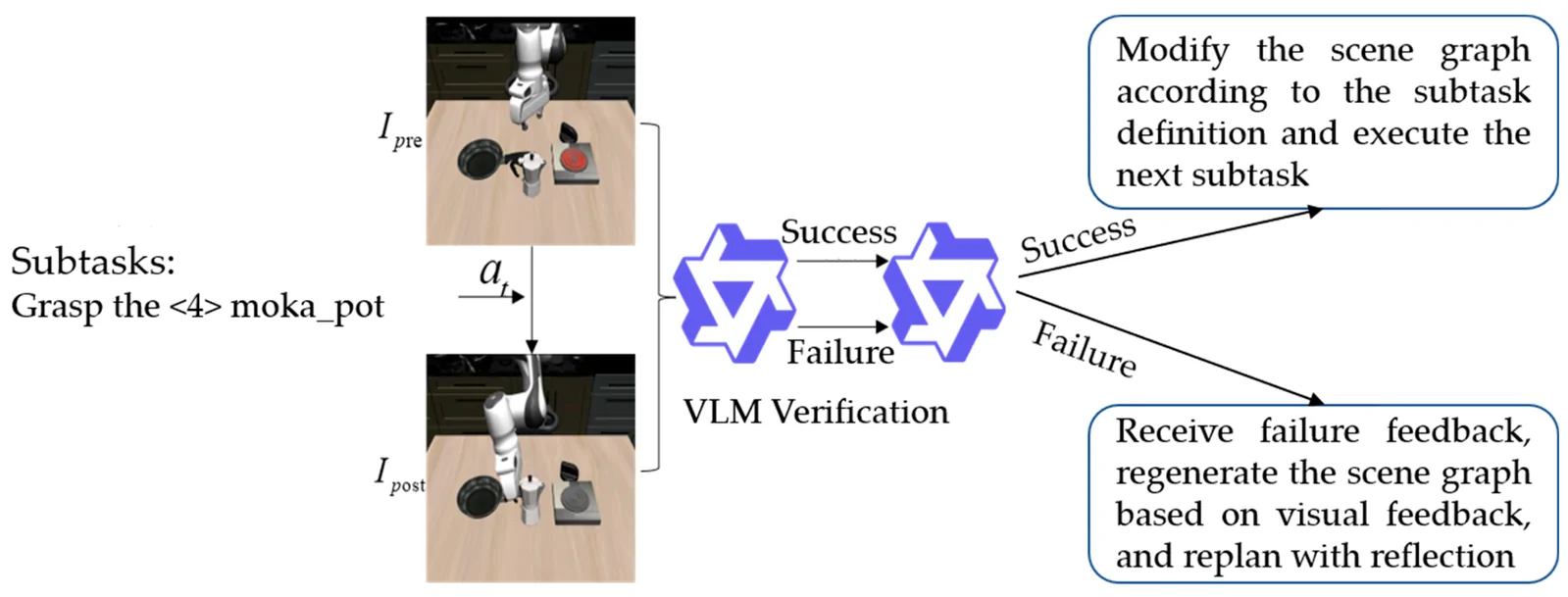
Dual-track closed-loop feedback mechanism based on visual expectations.

Although the generated initial subtask sequence $A_{t}$ is logically complete, the physical execution suffers from substantial randomness. To guarantee stable deployment from high-level planning to low-level control while addressing task failures caused by grasp slippage, unexpected object displacement, accidental collisions, and other anomalies. The system establishes a dual-track closed-loop feedback mechanism based on visual expectations, as illustrated in Figure 8. At the execution level, drawing on prior work, the planning system constructs a universal manipulation skill mapping interface is constructed. This interface maps skill-primitive-based manipulation commands $a_{t}$ output by the high-level planner to the low-level atomic skill library, which controls joint motions to accomplish task execution. To realize closed-loop automatic correction and recovery upon failed subtask execution, the system introduces the VLM as an execution validator, which maps execution outcomes back to the semantic space for verification. Let $I_{pre}$ and $I_{post}$ denote the image states before and after the low-level actuator executes a subtask manipulation $a_{t}$, respectively. Leveraging the VLM, the validator compares the semantic differences between the two image frames to infer and calculate whether the state transition conforms to the target state upon completion of the subtask manipulation $a_{t}$, and outputs a Boolean verification signal β indicating whether the subtask is finished. β can be formulated as follows:

(1) $$\beta =\mathrm{Verify}(I_{pre},I_{post},a_{t})\in \{\mathrm{True},\mathrm{False}\},$$

To mitigate hallucination risks and enhance the reliability of VLM-based verification, four complementary design strategies are embedded in the verification pipeline:

First, the verification task is framed as constrained binary state comparison rather than open-ended generation. Unlike the planner, which synthesizes complete action sequences from abstract instructions, the verifier only judges whether the post-execution state matches the predefined postconditions of the current atomic primitive. This substantial reduction in task complexity inherently improves the stability and accuracy of VLM outputs.

Second, verification prompts incorporate structured checklists tailored to each primitive type. For example, when verifying a “Place” action, the prompt explicitly directs the VLM to inspect three criteria: gripper openness, object arrival at the target position, and post-placement stability. This guided attention mechanism reduces ambiguous judgments and focuses the model on task-critical visual features.

Third, the dual-track closed-loop architecture provides implicit cross-validation. When semantic verification reports success, the system applies rule-based local updates to the scene graph. If subsequent planning detects unexplainable state inconsistencies, a full scene graph reconstruction is triggered as a fallback. This hierarchical design prevents isolated verification errors from propagating through the task pipeline.

Finally, the verifier shares the same zero-temperature inference configuration as the planner (temperature = 0), eliminating stochastic output variability and ensuring deterministic, reproducible judgments. While this does not eliminate hallucinations entirely, it renders error patterns consistent and diagnosable.

If the verification passes (β=True), it indicates that the executed manipulation has completed the corresponding subtask, and the real scene state is theoretically consistent with the internal expected state. To guarantee smooth continuous manipulation and high-frequency response of the robot, the system avoids re-invoking resource-intensive perception modules to scan and perceive the full scene or reconstruct the entire scene graph. Instead, it adopts an observation-free updating mechanism based on semantic postconditions. Specifically, the system rapidly modifies the scene graph $G_{t+1}$ locally directly according to the definition of the current manipulation primitive: for spatial transition operations involving position changes (e.g., grasping, placing), the topological connections between nodes are updated by removing outdated edges and creating new ones; for operations causing state variations (e.g., opening, closing), the attribute labels of the corresponding nodes are modified directly. This mechanism maintains consistency between the scene graph and the real environment through fast local revisions and drastically reduces computational overhead. This rule-based state update process can be formulated as follows:

(2) $$G_{t+1}\leftarrow {\mathrm{Update}}_{logic}(G_{t},a_{t}),$$

When verification fails (β=False), it indicates that deviations occurred during subtask execution and led to execution failure. At this moment, the state of the task scene has changed and fails to meet the sub-goal expectations, simple logical rule-based updates no longer work. The system therefore needs to perceive the current scene, update the scene graph, and regenerate a new task plan. First, the system triggers visual-semantic diagnosis. In this process, the VLM acts as a “failure analyzer” to conduct an in-depth comparison of semantic differences between pre-execution image state $I_{pre}$ and post-execution image state $I_{post}$, infer the root causes of task failure (such as grasp position offset or execution collision), and generate corresponding failure feedback $F_{err}$. This failure feedback is injected as a negative constraint into the planner’s historical context to reduce the probability of the system repeating the same mistakes in subsequent reasoning. Afterwards, to eliminate cumulative errors in state estimation, the system forcibly invokes the low-level perception model to perform a full scan and update of the current environment. It re-derives the positional relations of all objects and reconstructs a new scene graph to replace the outdated one. This process can be formulated as follows:

(3) $$G_{t+1}\leftarrow \mathrm{Perception}(I_{post}),$$

Finally, the VLM performs replanning based on the latest scene graph $G_{t+1}$ and contextual prompts containing failure lessons $F_{err}$. Instead of simply repeating the incomplete subtask, the model adaptively generates a new task plan with remedial strategies by analyzing and understanding the root causes of failures. This endows the robot with the capability to autonomously recover from uncertain errors.

## 4. Results and Discussion

To comprehensively evaluate the proposed task planning method ATP-VPDSG, a series of experiments were conducted in the LIBERO simulation environment [25]. For the generated subtask sequences, the proposed method realized low-level robot control through a set of predefined atomic skills, which were implemented using the APIs provided by the benchmark environment.

### 4.1. Experimental Setup

#### 4.1.1. Simulation Environment

**Figure 9:**
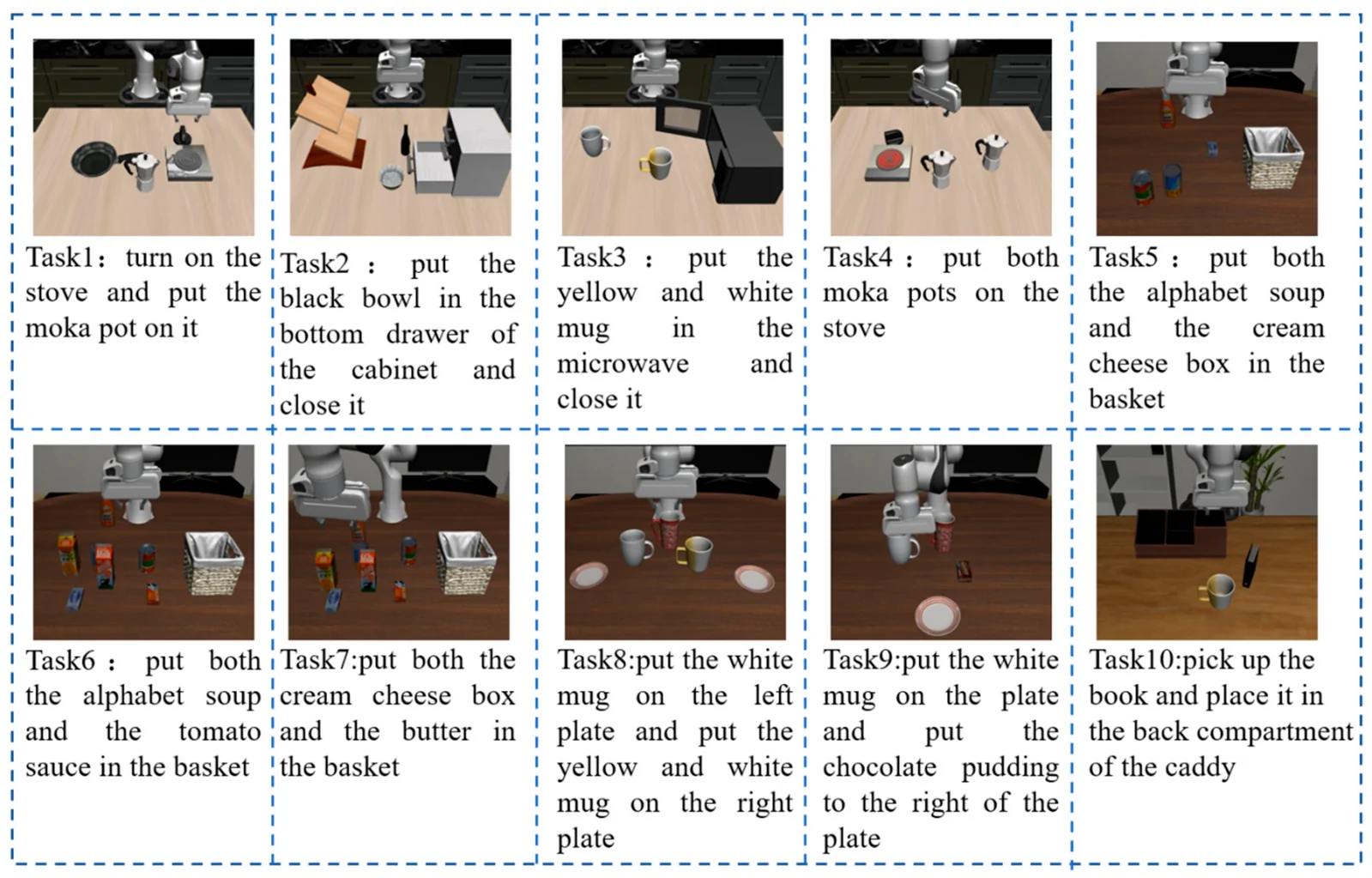
Task scenarios and detailed task descriptions of LIBERO-10.

In this work, the LIBERO benchmark was selected as the simulation platform. Built on the MuJoCo physics engine, LIBERO is capable of simulating complex object dynamics and high-fidelity physical interactions. In terms of specific task selection, the experiments focused on the challenging LIBERO-10 task subset, which consists of ten carefully designed long-horizon manipulation tasks. These tasks simulate complex scenarios in household kitchens, living rooms, and studies, as illustrated in Figure 9. In these scenarios, the robotic manipulator is required to perform multi-step interactions with hinged objects such as drawers and cabinet doors, as well as rigid objects including tableware and bottles. Subtle adjustments are made to the layout of objects in each evaluation trial, which enables verification of the generalization capability of the proposed method.

Unlike short-horizon tasks that mainly focus on single-step manipulation, the core challenge of LIBERO-10 lies in multi-stage hybrid manipulation and strict causal dependencies. Each task contains more than five action steps with explicit causal dependencies. Taking the representative Task 2 as an example, the task instruction is “place the black bowl into the bottom drawer of the wooden cabinet.” This task cannot be accomplished through a single pick-and-place operation. Instead, the robot must first determine whether the drawer is open. Only after satisfying this physical prerequisite can subsequent operations, including moving, grasping, placing, and closing the drawer, be executed. Any incorrect planning step by the planner may result in task failure. Furthermore, the demonstration data provided by this environmental benchmark can be utilized by the proposed method to extract task priors. Therefore, this task subset naturally aligns with the requirements for validating the proposed planning method.

#### 4.1.2. Core VLM and Its Configuration

The proposed task planning system adopted Qwen3-VL as its decision core VLM [26,27]. Qwen3-VL demonstrates significant improvements in high-resolution dynamic visual perception and complex instruction following. Compared with its predecessors, it provides more accurate parsing of structured scene descriptions and stronger long-window context memory, which are critical for long-sequence reasoning involving multi-object state variations such as video prior extraction and task planning. To satisfy the stringent requirements of robotic task planning for deterministic and reproducible outputs, a rigorous parameter configuration strategy is adopted. First, the inference temperature is fixed at 0 to completely suppress the randomness of large model generation, eliminate potential visual hallucinations in complex interaction scenarios, and guaranteeing fully reproducible planning results under identical environmental inputs. Second, the maximum generation length is set to 4096 to accommodate the output length demand for complete CoT reasoning in long-horizon tasks. All experiments were implemented in Python 3.10 with PyTorch 2.1, Transformers 4.40, and Grounding DINO v2.

### 4.2. Evaluation Experiments and Performance Analysis

#### 4.2.1. Manipulation Prior Extraction Experiments

**Figure 10:**
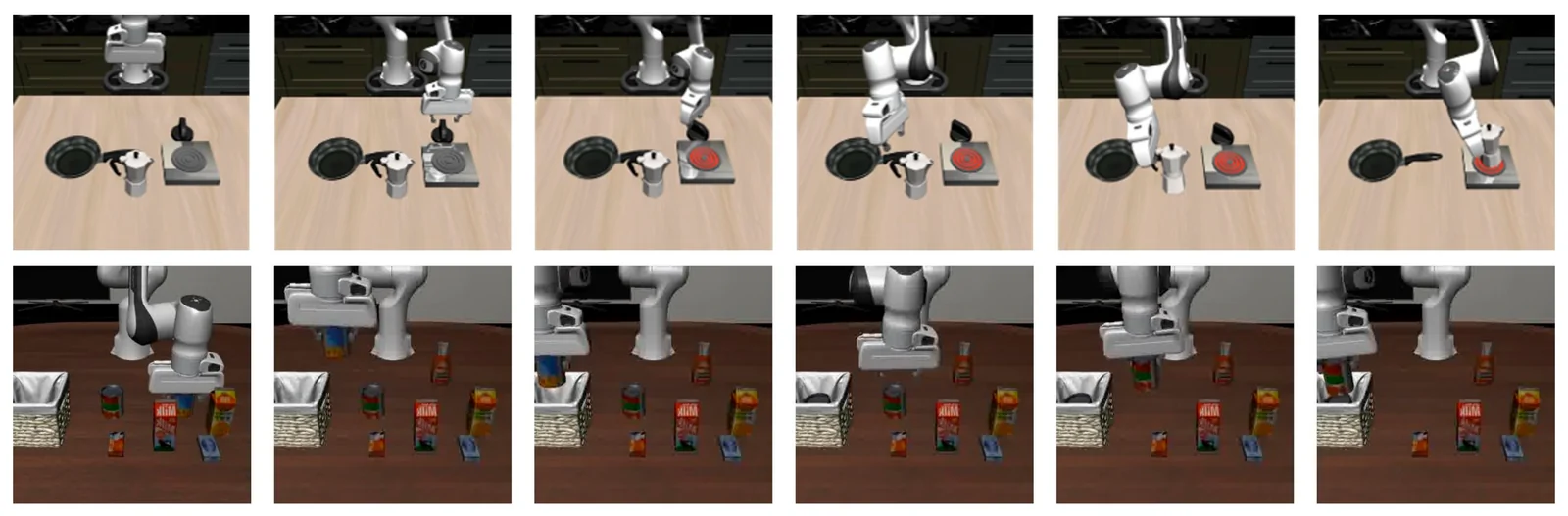
Execution sequences of LIBERO-10.

To verify the generalization capability of the proposed manipulation prior extraction module, we conduct systematic parsing experiments on all ten tasks of the LIBERO-10 benchmark, with keyframe compression ratio and prior knowledge parsing accuracy adopted as the core quantitative evaluation metrics. To intuitively demonstrate the extraction pipeline and structured output format, Task 1 and Task 6 are selected as representative examples for detailed illustration, whose execution sequences are presented in Figure 10. Task 1, “turn on the stove and put the moka pot on it”, involves relatively high manipulation difficulty: the robotic manipulator is required to first move above the stove switch, grasp and rotate the switch to activate the stove, release the gripper, then move above the moka pot, grasp and transport the pot to the stove surface, lower and release the pot, and finally retract the manipulator. Task 6, “put both the alphabet soup and the tomato sauce in the basket”, is a typical multi-object pick-and-place task, in which the manipulator needs to sequentially complete the grasping, lifting, transporting and basket-placing of two cans while avoiding already placed objects and surrounding obstacles, thus posing clear requirements on operation sequencing and spatial positioning.

**Figure 11:**
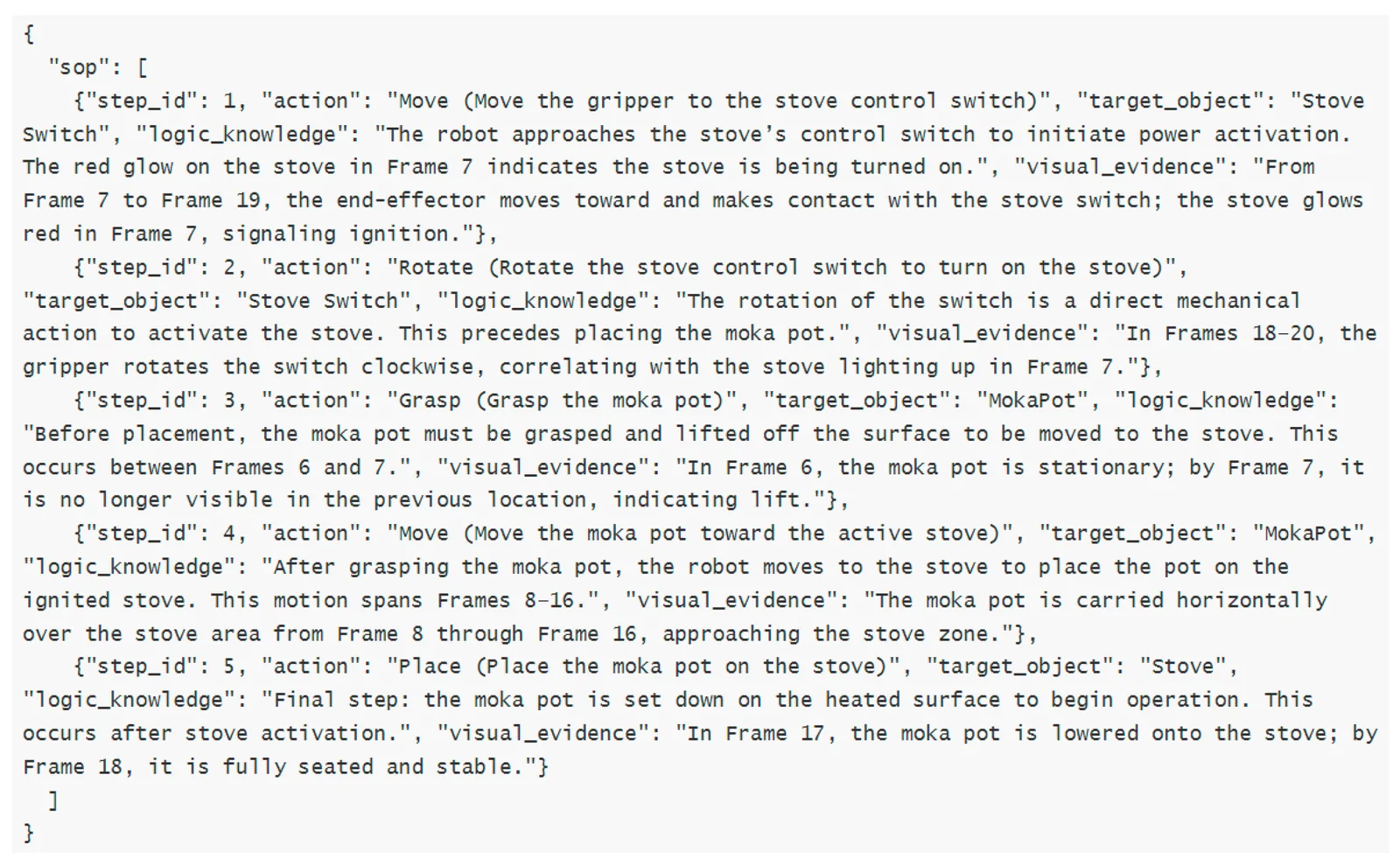
Structured manipulation knowledge extracted from video demonstrations.

Figure 11 presents the structured storage of the extracted manipulation knowledge. It mainly consisted of three components: the manipulation sequences to complete the task, the execution logic of each manipulation step, and corresponding visual evidence. The extracted manipulation sequences included moving the gripper to the stove control switch, rotating the control switch to turn on the stove, grasping the moka pot, moving the moka pot toward the active stove, and placing the moka pot on the stove. The obtained manipulation sequences were generally consistent with the standard manipulation workflow of Task 1.

By analyzing the extracted manipulation knowledge, it was observed that although the manipulation logic and execution conditions generated by the VLM may contain partial deviations, the overall parsing accuracy remained high due to its powerful reasoning and spatial perception capabilities. Therefore, it was fully feasible to leverage the VLM to analyze video key frames and distill manipulation workflows as well as conditional priors. After structural processing, the extracted knowledge could be fed into the VLM planner as contextual information to provide reliable logical references for subsequent task planning. The results verified the effectiveness of the proposed video prior extraction module, which could extract transferable manipulation knowledge from demonstration trajectories and improve the reasoning performance of VLM-based robotic planner.

To further quantify the generalization and robustness of the extraction module, we conducted full-task parsing experiments on all ten LIBERO-10 tasks, along with robustness tests under noisy and low-frame-rate settings.

Table 2 summarizes the performance across all tasks. The adaptive SSIM sampling strategy consistently compresses raw demonstration videos by 60–75% while retaining all critical state transitions for different task types. The parsing accuracy of manipulation sequences ranges from 85.3% to 96.0%, with an average of 91.2% across all tasks. Tasks involving subtle state changes (e.g., knob rotation and lid closing) show relatively lower accuracy, as fine-grained motion transitions are harder to distinguish from inter-frame differences.

#### 4.2.2. Planning Accuracy

**Figure 12:**
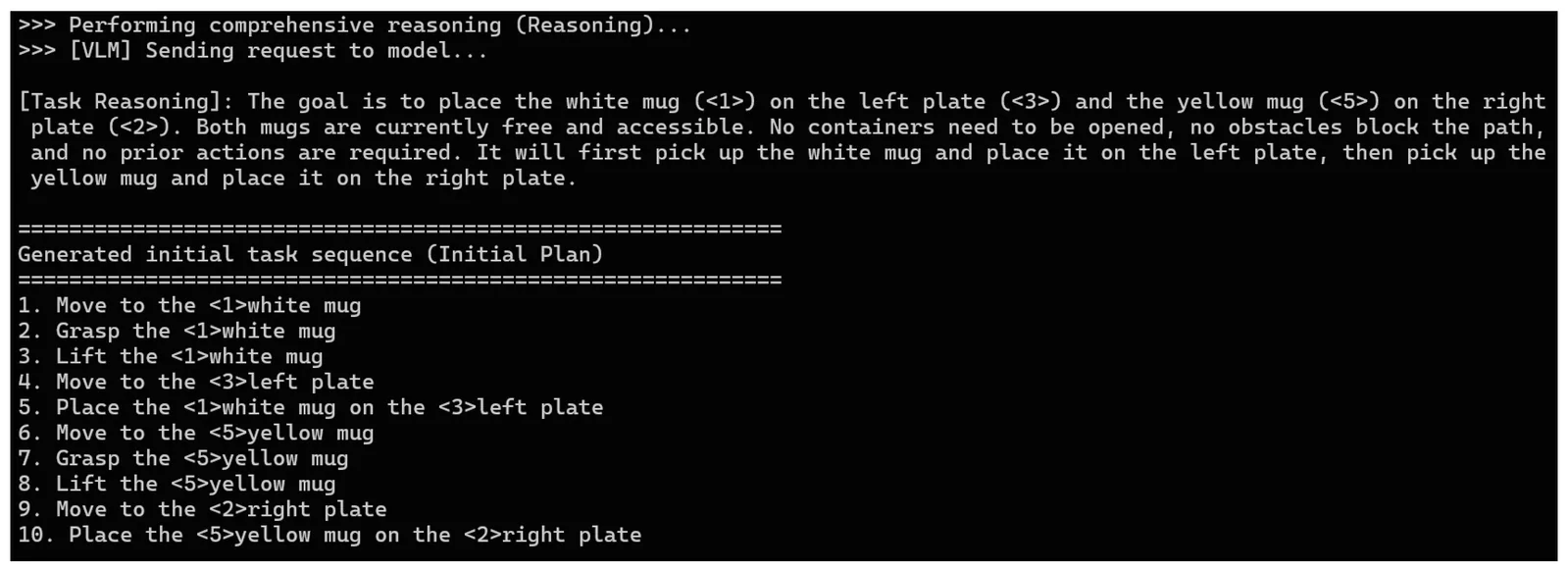
Planning results of Task 8.

To evaluate the preliminary planning performance of the proposed planning method ATP-VPDSG, this study conducted task planning validation on all ten benchmark tasks. Fifty planning outputs of each task were selected for analysis and evaluation. The task planning accuracy rates are presented in Table 2. The average accuracy across all ten tasks reaching 91.2%. Among all tasks, Task 2, Task 3 and Task 9 achieved relatively lower success rates. Further analysis revealed that planning failures mainly stem from inaccurate extraction of prior knowledge by the VLM and unreliable discrimination of subtle object states. Task 8 achieved the highest planning accuracy. The planning results of Task 8 are illustrated in Figure 12. The scene graphs built upon visual prompting assigned a unique ID to each object and recorded their relative positional relations. This mechanism facilitates the spatial reasoning capacity of the VLM and improved overall task success rates.

#### 4.2.3. Failure Diagnosis Accuracy

To quantitatively evaluate the failure diagnosis performance of the dual-track verification mechanism, we collected and manually annotated all failure instances generated in the complete experiments, and categorized them into four typical types: grasp slippage, object position confusion, collision with obstacles, and occlusion interference. The classification accuracy of the VLM-based failure analyzer on each error type is summarized in Table 3.

The failure analyzer achieves an overall classification accuracy of 87.3%. Collision failures obtain the highest accuracy due to their visually distinctive features of contact and displacement. Occlusion-related failures are the most challenging, as partial object visibility makes root cause attribution inherently ambiguous and prone to misjudgment.

From the mechanism perspective, the dual-track design has two inherent advantages over existing failure reflection methods. First, the embedded video prior provides the analyzer with an explicit expected procedure reference, enabling targeted root cause diagnosis rather than generic post hoc explanation. Second, the structured scene graph supplies deterministic state information that narrows the hypothesis space of failure causes, reducing ambiguous reasoning. A systematic quantitative comparison against baseline methods under a unified benchmark will be conducted in future work.

### 4.3. Comparative Experiments

In the comparative experiments, the proposed method ATP-VPDSG was evaluated against three selected VLM-based robotic task planning baselines across all ten tasks in the LIBERO-10 task subset. The compared baselines are detailed below.

1. VILA [11]: A VLM-based robotic long-horizon task planning method that directly integrates visual observations and language instructions for joint reasoning, generating executable subtask sequences, without requiring external adaptation modules or VLM fine-tuning;
2. ReplanVLM [13]: A VLM-based robotic task planning framework centered on full-process error control combining pre-execution error prevention and post-execution error correction. The coordination of these two mechanisms improves the robustness and accuracy of task execution in open-world environments;
3. Prompt2Act [14]: A robotic task framework with a VLM as its core planning engine. It innovatively integrates semantic visual augmentation and dynamic hybrid execution strategy. This design addresses the drawbacks of conventional VLM planning, including ambiguous fine-grained localization and monotonous execution policies. It realizes accurate and efficient mapping from multimodal prompts to robotic action sequences, making it suitable for complex manipulation scenarios in the open world.

During the experiments, each planning method was evaluated 30 times over the provided ten tasks. At the execution level of the proposed method, the planned subtask actions were mapped to predefined atomic skills implemented via the APIs provided by the benchmark environment. The positional information of manipulated objects was retrieved through privileged information interfaces provided by the environment. In the task evaluation phase, the planning method repeatedly invoked predefined atomic skills to perform manipulations, with a maximum execution step limit set to 600 timesteps. Finally, the benchmark environment judged task success based on its privileged information.

The performance of VILA, ReplanVLM, Prompt2Act, and the proposed ATP-VPDSG on the LIBERO-10 tasks is presented in Table 4. Methods such as VILA, ReplanVLM, and Prompt2Act directly adopted VLMs for planning but lack manipulation knowledge of relevant objects. Therefore, they delivered inferior performance on tasks that demand intensive object interaction, including Task 1, Task 2, and Task 3. In particular, all three baseline methods achieved a 0% success rate on Task 1, which required turning on the stove and placing the moka pot on it. Furthermore, VILA and ReplanVLM relied purely on raw image inputs for scene observations and suffered from limited perception of spatial layouts, leading to inaccurate planning and consequently mediocre performance on long-horizon pick-and-place tasks.

The proposed ATP-VPDSG extracted and embedded manipulation prior knowledge to introduce logical constraints for object interactions. Meanwhile, it leveraged vision-prompted image inputs as well as spatial structure representations derived from scene graphs. Experimental results demonstrated that ATP-VPDSG achieved superior performance, with the overall average success rate improved by approximately 16.67%. ATP-VPDSG outperformed all baseline methods on nine out of ten tasks and was only slightly inferior to Prompt2Act on Task 8. It was noteworthy that Prompt2Act also adopted a vision prompting annotation strategy similar to ATP-VPDSG. The key distinction lain in its reliance on SAM to segment object regions, then overlaid object names and central coordinates onto the corresponding original image regions. This implementation was more complex than the proposed method.

To further understand the remaining failure modes, we conducted a detailed root-cause analysis on the 25.33% unsuccessful task executions. All failure instances were manually reviewed and categorized into three classes: (i) low-level physical execution errors, (ii) perceptual misclassifications in scene graph generation, and (iii) high-level planning logic errors. The distribution is summarized in Table 5.

Low-level execution errors account for the largest share, primarily caused by imperfect grasp pose estimation and object displacement during transport. In these cases, the high-level plan was correct, but atomic skill execution failed due to control-level imprecision. Perceptual misclassifications mainly involve subtle state judgments under occlusion or ambiguous visual cues. Planning logic errors, which the proposed framework directly targets, account for the smallest share, indicating that video priors and scene graph constraints effectively reduce cognitive-level errors. This analysis suggests that the primary remaining bottleneck lies in low-level execution reliability, motivating future work on integrating more robust grasp planning and compliant control strategies.

### 4.4. Ablation Studies

To verify the contribution of each functional component to overall performance, this study evaluated the execution performance of the system across the ten test tasks by removing specific modules. The ablation studies consisted of two comparative configurations: without demonstration video priors, and without visual markers and scene graphs, as detailed below.

1. Without demonstration video priors: The VLM only received high-level task instructions and current observations. The standardized manipulation sequences extracted from video demonstrations were not fed into the prompt as prior knowledge, forcing the VLM to perform planning relying solely on its inherent zero-shot capability;
2. Without visual markers and scene graphs: Raw RGB images were input to the VLM with overlaid ID labels and semantic markers removed, and no JSON-formatted scene graphs constructed. Only natural language descriptions of objects were provided within the prompts.

**Figure 13:**
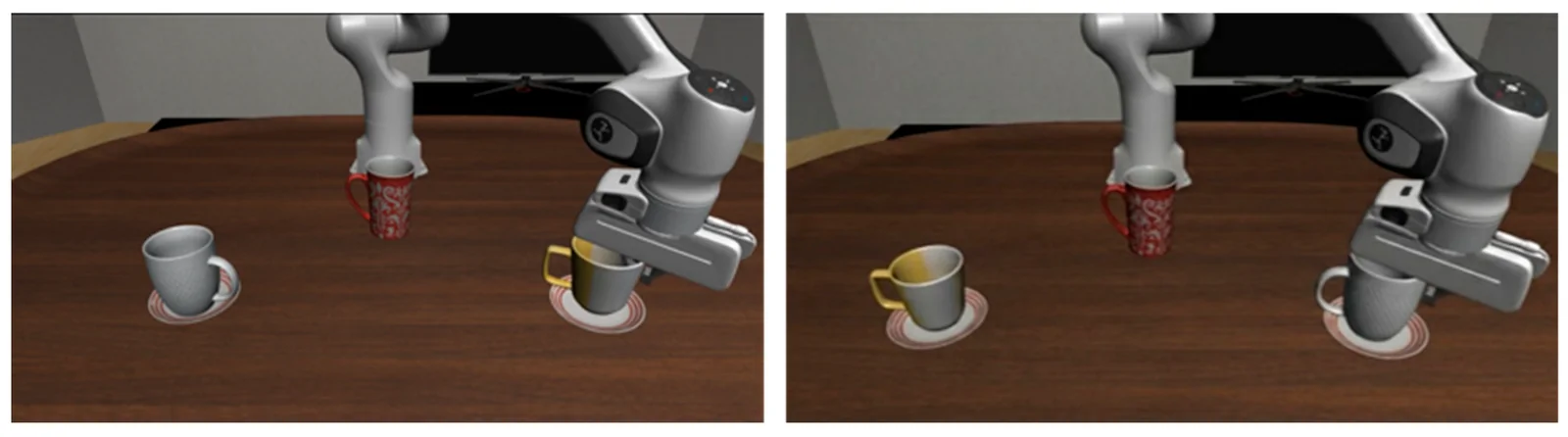
Failure case in Task 8 without visual markers and scene graphs: the positions of the two cups are swapped.

The results of ablation studies are listed in Table 6. The results revealed that the average success rate across the ten tasks dropped by 9.34% after removing demonstration video priors. This demonstrated that relying merely on the pre-trained commonsense knowledge of the VLM was insufficient for planning complex interactive manipulations. In addition, eliminating visual markers and scene graphs led to a 10.34% decline in average success rate. Such omission drastically impaired the model’s comprehension of intricate spatial layouts and readily triggered erroneous judgments of spatial relations, for instance, the positions of the two cups were swapped in Task 8, as shown in Figure 13. It should be emphasized that the following analysis of individual scene-graph sub-components is qualitative, based on failure case inspection rather than separate quantitative ablation experiments. Such qualitative analysis provides preliminary insights into their respective roles, while a comprehensive fine-grained quantitative ablation is left for future work. When the full scene graph module is removed, the dominant failure mode is instance reference confusion, indicating that unique ID annotation delivers the largest performance gain by resolving referential ambiguity. Misjudgments of spatial containment and relative positioning also account for a notable proportion of failures, suggesting that topological relation parsing further improves performance by supplying explicit geometric constraints. Grounding DINO yields more stable open-vocabulary detection than the VLM’s native perception, reducing missed and false detections. A comprehensive fine-grained quantitative ablation will be conducted in future work to systematically validate each sub-component’s individual contribution. Ablation studies demonstrated that demonstration video priors and dynamic scene graphs exert complementary effects on logical constraints and physical feasibility.

### 4.5. Computational Overhead and Real-Time Performance

To evaluate the engineering deployability of the proposed method, we statistically analyzed the inference latency of each core module on a single NVIDIA GeForce RTX 5090 (32 GB) GPU. The computational overhead is divided into offline one-time overhead and online runtime overhead, as summarized in Table 7. Note that the reported latency values measure VLM inference time only and do not include image pre-processing overhead (e.g., resizing, normalization, and object detection via Grounding DINO).

The video prior extraction module runs completely offline: it only needs to be executed once when a new task type is introduced, and the extracted structured priors can be reused for all subsequent instances of the same task, so it does not affect online runtime performance. For online execution, initial scene graph construction and full-sequence planning are completed once at task startup, with a total overhead of approximately 4 s. The most frequently executed single-step verification maintains latency below 1 s.

For task-level robotic manipulation, the proposed multi-stage reasoning pipeline satisfies the real-time requirements of discrete operation scheduling. Since low-level motion control is handled by the atomic skill layer at a much higher frequency, the planning layer does not require millisecond-level response. For larger environments with more objects, scene graph construction latency grows gently with instance count, and VLM inference latency increases linearly with context length. Performance can be further optimized via context window cropping, parallel scene graph parsing, and lightweight VLM distillation to support larger-scale open-world scenarios.

It is worth noting that the proposed framework is inherently model-agnostic. All components interact with the VLM through standard multimodal prompts without any model-specific fine-tuning or architecture-dependent modules. The ablation study in Section 4.4, in which all variants share the same backbone and prompting protocol, isolates the contribution of each proposed component from the intrinsic capability of the VLM itself.

### 4.6. Generalization to Out-of-Distribution Tasks

A natural concern regarding video priors is the risk of overfitting to demonstrated task topologies, which may limit performance on out-of-distribution (OOD) tasks. We analyze this issue from three perspectives.

First, for geometric variations within the same task category, the LIBERO-10 benchmark randomizes object positions and initial poses across each trial. The consistent average success rate of 74.67% across 30 trials per task demonstrates that the framework is robust to layout-level distribution shifts, as the scene graph provides instance-level spatial grounding that adapts to each new configuration.

Second, for tasks that share sub-skills with demonstrated tasks but involve different objects, the hierarchical structure of the extracted priors enables partial transfer. The prior knowledge is organized as sequences of parameterized primitives (e.g., “grasp–transport–place on heating element”) rather than object-specific trajectories. When a novel task shares primitive-level structure with a demonstrated task, the retrieval module can return partially relevant priors as soft references, while the scene graph provides task-specific spatial constraints. This compositional reuse of sub-skills mitigates the overfitting risk at the topology level.

Third, for completely novel tasks with no relevant priors, the framework is designed to degrade gracefully rather than fail catastrophically. When the prior retrieval module returns empty or low-confidence results, the planner automatically falls back to zero-shot VLM reasoning with scene graph constraints, which is equivalent to the “w/o video prior” ablation variant (58.33% average success rate). This ensures that the inclusion of video priors never hurts performance on OOD tasks: the system benefits from priors when relevant ones exist and reverts to baseline capability when they do not.

A systematic quantitative evaluation on dedicated OOD benchmarks, including cross-task and cross-domain transfer settings, is left for future work.

### 4.7. Real-Robot Experiment

To further validate the effectiveness of the proposed method in real-world scenarios, we conducted a real-robot experiment on an industrial slider–rail assembly task, which is a typical long-horizon, high-precision assembly procedure involving grasping, alignment, insertion, and pushing operations.

**Figure 14:**
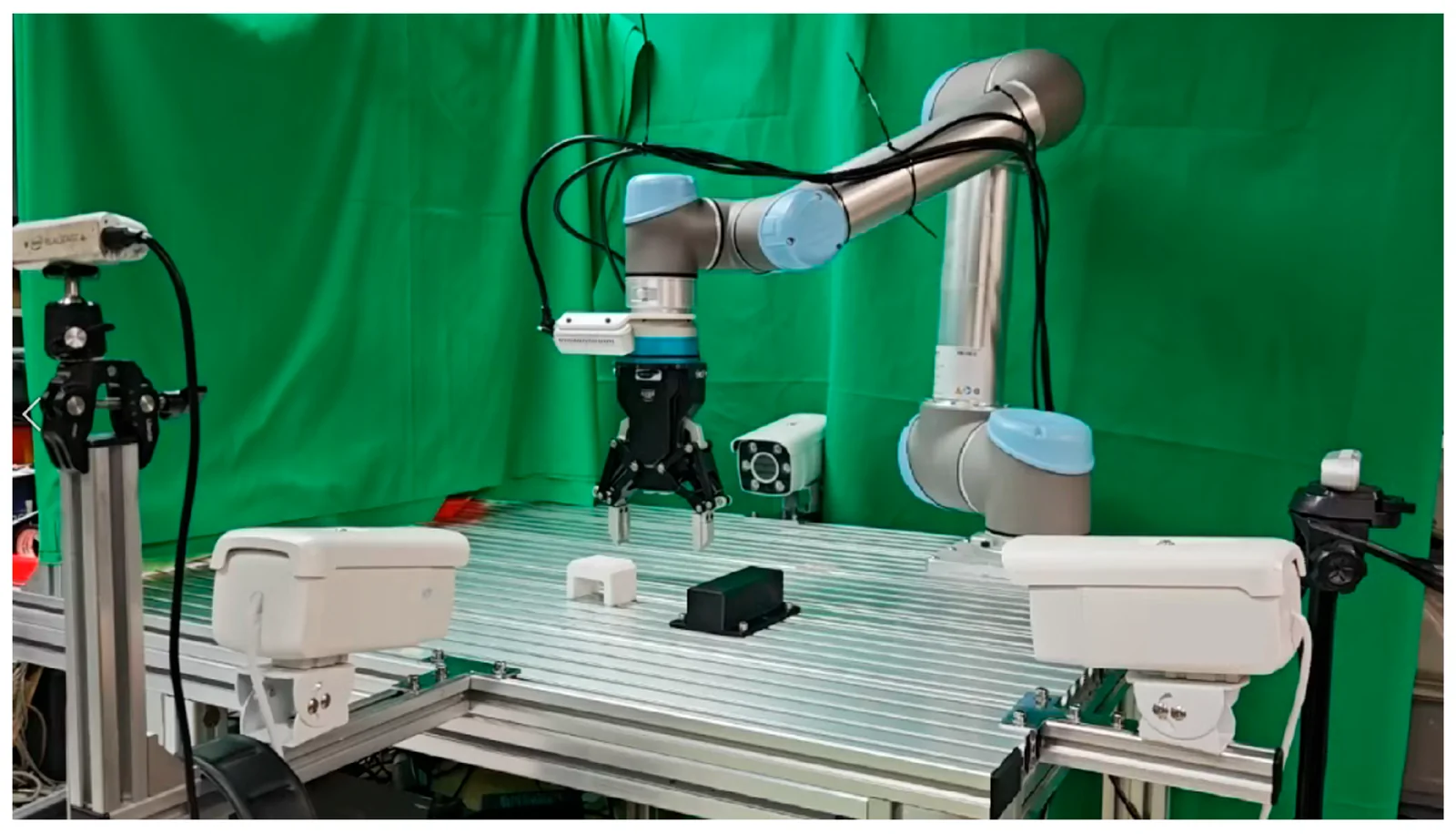
Real-robot experimental platform for the slider–rail assembly task.

Hardware setup. The experimental platform is shown in Figure 14. A 6-DOF robotic arm equipped with a parallel gripper was used as the execution platform. An RGB camera was mounted at a fixed external viewpoint to capture workspace observations. The linear rail is approximately 200 mm long and 35 mm wide, with a fitting tolerance of 2 mm between the slider and the rail. The slider was placed at random positions on the workbench in each trial, reproducing the industrial challenge of randomized workpiece placement.

**Figure 15:**
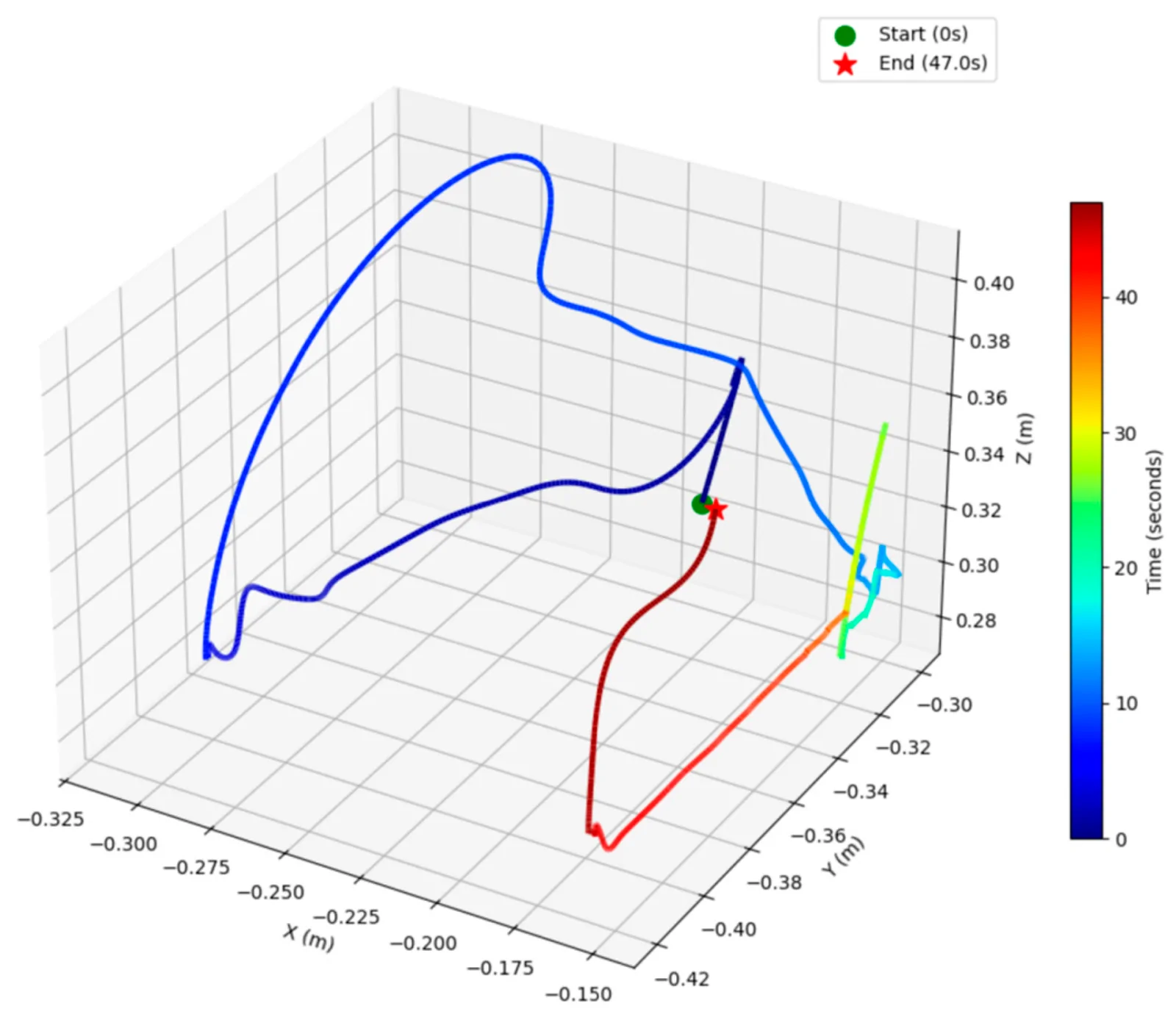
End-effector execution trajectory for the slider–rail assembly task.

**Figure 16:**
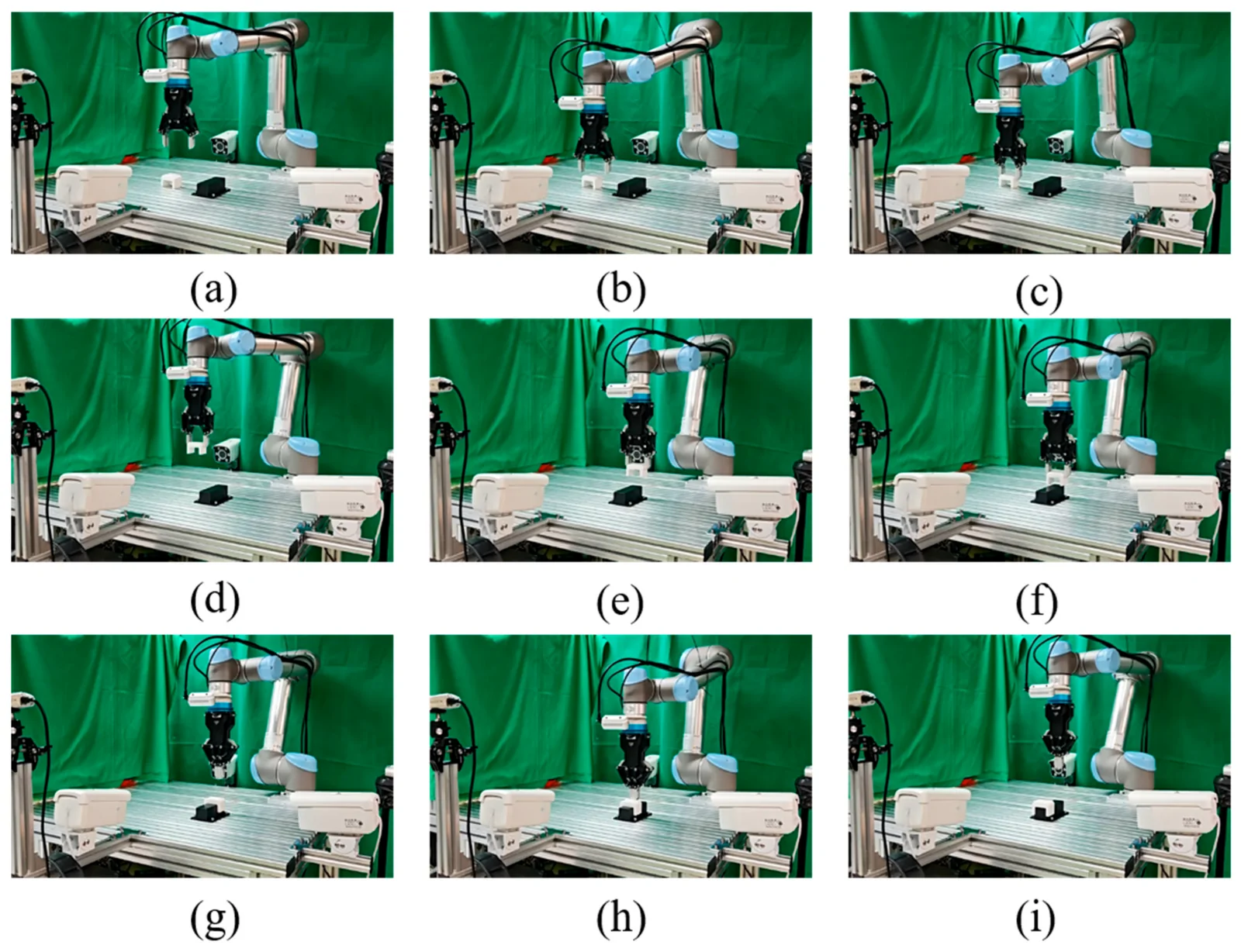
Successful execution sequence of the slider–rail assembly. (**a**) Initial pose with the slider placed randomly on the workbench; (**b**) moving the end-effector above the slider; (**c**) adjusting the end-effector pose for grasping; (**d**) grasping and lifting the slider; (**e**) transporting the slider above the installation end of the rail; (**f**) aligning the slider with the rail; (**g**) pressing down to mount the slider and releasing the gripper; (**h**) pushing the slider along the rail to the installation end; (**i**) returning to the initial pose upon task completion.

Task description. The task requires the robot to complete a multi-step assembly procedure: (1) identify the randomly placed slider and move the end-effector above it; (2) adjust the end-effector pose and grasp the slider; (3) lift the slider and move it above the installation end of the rail; (4) align the slider with the rail; (5) press down to mount the slider onto the rail and release the gripper; (6) push the slider along the rail to the installation end; and (7) return to the initial pose. The execution trajectory is illustrated in Figure 15, and a successful assembly sequence is shown in Figure 16.

Experimental setup. Fifty demonstration videos of the slider–rail assembly task was collected via a teleoperation device. To evaluate the sample efficiency of the proposed framework, two groups of experiments were conducted using 30 and 50 demonstration videos for video prior extraction, respectively. During data collection, the positions of both the slider and the rail were randomized to encourage spatial generalization. At the evaluation stage, 50 repeated trials were performed for each group, with workpiece positions randomized in every trial. The proposed ATP-VPDSG framework was applied without any task-specific fine-tuning: the video prior extraction module processed the demonstration videos to obtain structured standard operating procedures (SOPs), the dynamic scene graph was constructed from real-time RGB images, and the VLM planner generated sub-goal sequences that were executed via pre-defined atomic skills.

Results. The results are summarized in Table 8. With 30 demonstration videos, the system successfully completed 32 out of 50 trials (64.0%), with an average execution time of 21.32 s for successful runs. With 50 demonstration videos, the success rate increased to 82.0% (41/50), with an average execution time of 18.32 s.

These results demonstrate that the proposed task planning framework successfully transfers from simulation to a real industrial assembly scenario. The high-level planner decomposes the long-horizon assembly task into atomic sub-goals guided by video priors, while the dynamic scene graph provides accurate spatial grounding under randomized workpiece positions. Notably, even with only 30 demonstration videos—a 40% reduction—the system still maintains a 64.0% success rate without catastrophic performance degradation. This indicates that decomposing long-horizon tasks into primitive-level sub-goals substantially reduces the learning difficulty of each step, enabling few-shot task acquisition. The remaining failures were primarily caused by grasp pose misalignment and slider–rail insertion errors under tight tolerance, which are low-level control issues rather than planning errors. Future work will integrate execution prediction and compliant control strategies to further improve robustness.

## 5. Conclusions

### 5.1. Summary

This work investigated a VLM-based task planning method, aiming to address the logical reasoning and execution operations in complex task scenarios, improving the operational efficiency and accuracy of robots in complex multi-stage tasks. ATP-VPDSG was proposed to tackle the dual challenges of logical reasoning deviation of large pre-trained models in complex task planning and the lack of spatial geometric perception in unstructured environments, by integrating video demonstration priors and scene graphs to guide the VLM to generate task execution sequences that comply with logical constraints and physical feasibility. Furthermore, a dual-track closed-loop feedback mechanism based on visual expectations was introduced. Through execution verification, failure reflection and dynamic replanning, the robots were endowed with the ability of error correction and stable execution in complex environments. Experimental verification was carried out on the LIBERO-10 tasks combined with predefined atomic skills. The experimental results showed that the average accuracy of the proposed method in initial planning reached 91.2%, and the success rate of task execution combined with atomic skills reached 74.67%, which proved the effectiveness of the method. Comparative experiments demonstrated clear performance advantages over selected VLM-based baseline methods. Ablation studies further verified that video priors and dynamic scene graphs exert complementary effects on logical constraints and physical feasibility.

The proposed method ATP-VPDSG realizes the adaptive decomposition and reasoning for complex tasks by the VLM, effectively improves the success rate and robustness of robot task planning under dynamic environmental changes, and may yield useful guidelines for developing autonomous planning systems of embodied intelligent robots. Furthermore, the modular architecture of ATP-VPDSG makes it a promising candidate for industrial Robot-as-a-Service (IRaaS) deployment scenarios, where video priors could be maintained in a centralized cloud knowledge base and retrieved on demand by edge robots.

### 5.2. Limitations

The current method has several limitations. First, the current work only provides quantitative statistics of failure diagnosis accuracy on our own experimental data, and has not carried out a unified horizontal comparison with existing failure reflection methods under a standardized failure case benchmark. Second, the current method lacks the inherent predictive ability for the evolution of environmental states. Third, the current ablation study only validates the overall effect of the dynamic scene graph module, without fine-grained quantitative disentanglement of contributions from its internal sub-components. Additionally, the current experimental validation has three limitations: simulation experiments are limited to the LIBERO-10 benchmark, and although a real-robot pilot study on an industrial slider–rail assembly task is reported in Section 4.7, larger-scale real-world validation across diverse tasks is needed; the framework is evaluated with a single VLM backbone, and cross-model evaluation across different VLM families would provide further evidence; and evaluation on additional simulation benchmarks such as RoboCasa is needed to demonstrate broader cross-domain generality.

### 5.3. Future Work

Several directions are planned for future work. A dedicated benchmark for robotic manipulation failure diagnosis will be constructed to further verify the advantages of the dual-track mechanism. Systematic sub-component ablation experiments will be supplemented to further clarify the action mechanism of each module. Cross-model evaluation across different VLM families and evaluation on additional benchmarks such as RoboCasa will be conducted. Validating the framework on industrial assembly tasks and evaluating its scalability in multi-robot IRaaS ecosystems is an important direction. Future work will also focus on constructing a structured world model based on scene graphs, leveraging massive demonstration video data to learn the topological laws of state transitions of scene graph nodes and edges under action effects, rather than merely predicting pixel changes, so as to boost the robots’ ability to avoid deadlocks and adapt to dynamic environments in long-horizon task planning.

## Figures and Tables

**Table 1:** Defined basic skill primitives.

Skill Category	Basic Skill Primitives	Semantic Description
Spatial Motion	Move	Move the end effector above the target region
	Lift	Lift an object
Grasping and Placing	Grasp	Close the gripper to grasp an object
	Place	Open the gripper to release an object
Assembly and Adjustment	Align	Adjust pose for alignment
	Insert	Perform insertion
Interaction	Push	Apply pushing force to move an object, close a drawer, or remove an obstacle
	Pull	Apply pulling force while grasping, e.g., to open a drawer or cabinet door
	Rotate	Rotate a knob, valve, or similar part
	Press	Press a switch or button

**Table 2:** Video prior extraction performance across all LIBERO-10 tasks.

Task ID	1	2	3	4	5	6	7	8	9	10	Avg.
Video compression ratio	68%	72%	65%	70%	73%	71%	69%	67%	74%	72%	70.1%
Planning accuracy	92.0%	90%	88.0%	91.0%	94.0%	93.0%	91.0%	96.0%	85.3%	91.7%	91.2%

**Table 3:** Failure diagnosis accuracy across different error types.

Error Type	Grasp Slippage	Object Position Confusion	Collision with Obstacles	Occlusion Interference	Overall
Diagnosis accuracy	88.6%	90.2%	92.1%	79.5%	87.3%

**Table 4:** Success rates of different planning methods across the LIBERO-10 tasks.

Task	VILA	ReplanVLM	Prompt2Act	ATP-VPDSG
Task 1	0.00%	0.00%	0.00%	53.33%
Task 2	26.67%	23.33%	30.00%	56.67%
Task 3	33.33%	30.00%	36.67%	63.33%
Task 4	56.67%	53.33%	56.67%	73.33%
Task 5	63.33%	60.00%	73.33%	86.67%
Task 6	66.67%	63.33%	70.00%	86.67%
Task 7	60.00%	60.00%	66.67%	83.33%
Task 8	53.33%	50.00%	86.67%	80.00%
Task 9	66.67%	66.67%	80.00%	83.33%
Task 10	56.67%	60.00%	80.00%	80.00%
Avg.	48.33%	46.67%	58.00%	74.67%

**Table 5:** Root-cause distribution of the remaining failures.

Failure Category	Share of Failures	Share of Total Trials	Typical Cases
Low-level execution errors	45.2%	11.4%	Grasp slippage, object displacement during transport, collision due to trajectory deviation.
Perceptual misclassifications	24.7%	6.3%	Subtle state misjudgment (e.g., drawer partially vs. fully open), ID confusion under severe occlusion.
Planning logic errors	30.1%	7.6%	Incorrect prior retrieval, suboptimal replanning after repeated failures.

**Table 6:** Results of ablation studies.

Method	ATP-VPDSG	Without Demonstration Video Priors	Without Visual Markers and Scene Graphs
Success rate	74.67%	65.33%	64.33%

**Table 7:** Inference latency of each module.

Module	Latency (s)	Execution Mode	Execution Frequency
Video prior extraction	8.2–12.5	Offline	Once per task type
Initial scene graph construction	1.2–1.8	Online	Once per task instance
Full-sequence initial planning	2.5–3.2	Online	Once per task instance
Single-step visual verification	0.6–0.9	Online	Once per subtask
Failure diagnosis and replanning	2.1–2.8	Online	Triggered only on execution failure

**Table 8:** Real-robot slider–rail assembly results.

Demonstration Videos	Success Rate	Successful/Total	Avg. Success Time (s)
30	64.0%	32/50	21.32
50	82.0%	41/50	18.32

## Data Availability

The data supporting the findings of this study are available from the corresponding author upon reasonable request.
